# LINC00922 decoys SIRT3 to facilitate the metastasis of colorectal cancer through up-regulation the H3K27 crotonylation of ETS1 promoter

**DOI:** 10.1186/s12943-023-01859-y

**Published:** 2023-10-04

**Authors:** Meijian Liao, Xiaolin Sun, Wendan Zheng, Mengdi Wu, Yifan Wang, Jia Yao, Yu Ma, Shoucui Gao, Dongsheng Pei

**Affiliations:** Department of Pathology, Xuzhou Medical University, Xuzhou, 221004 P.R. China

**Keywords:** Colorectal cancer, Metastasis, LINC00922, H3K27cr, SIRT3

## Abstract

**Background:**

Lysine crotonylation (Kcr) is up-regulation in colorectal cancer (CRC) tissues, while its specific contribution remains uncertain. This study aimed to elucidate the role and mechanism of crotonylation on Lys27 of histone H3 (H3K27cr) in facilitating CRC metastasis.

**Methods:**

Immunohistochemistry was employed to investigate the correlation between H3K27cr and CRC metastasis. Both in vitro and in vivo assays employing loss function or gain function approaches were conducted to elucidate the role of LINC00922 in promoting CRC metastasis. ScRNA-seq analysis and immunoprecipitation analyses were employed to explore the underlying mechanism by which LINC00922 facilitates CRC metastasis through H3K27cr.

**Results:**

Clinically, H3K27cr was upregulated in metastatic CRC tissues and positively correlated with advanced clinical stages. Functionally, knockdown of LINC00922 inhibited migration of CRC cells both in vitro and in vivo. Furthermore, the supplementation of NaCr restored the migration and invasion levels of LINC00922 stable knockdown cells by restoring the H3K27cr level. Mechanistically, LINC00922 promoted invasion and migration through H3K27cr mediated cell adhesion molecules (CAMs) in epithelial cells. Notably, LINC00922 interacted with the protein sirtuin 3 (SIRT3) and obstructed its binding to the promoter region of ETS1, leading to an elevation in the level of H3K27cr in this promoter region and the subsequent activation of ETS1 transcription.

**Conclusions:**

Our findings uncovered a novel regulatory function of H3K27cr, regulated by LINC00922, in facilitating CRC metastasis. This discovery contributed to a deeper comprehension of the involvement of histone crotonylation in the metastatic process of CRC.

**Supplementary Information:**

The online version contains supplementary material available at 10.1186/s12943-023-01859-y.

## Introduction

A recently identified post-translational modification, known as lysine crotonylation (Kcr), has been observed in both human and mouse cells [1]. Kcr has been found to play critical roles in the differentiation of stem cells [2], spermatogenesis [3], acute kidney injury [4], HIV latency [5], autophagy [6], DNA repair [7], and cancer progression [8]. Crotonyl-CoA is the crotonyl donor during crotonylation and is essential in regulating intracellular crotonylation level. Compared with other tissues, the colon has the highest level of histone crotonylation [9]. This is probably due to short-chain fatty acids (SCFAs) derived from the gut microbiome contributing to high level of Crotonyl-CoA [10]. Moreover, the level of crotonylation modification is higher in colon cancer tissues than in the adjacent tissues. So far, it is still unclear why crotonylation modification is upregulated in CRC tissues. Furthermore, whether upregulated crotonylation contributes to occurrence and development of CRC is still poorly understood. Only a few studies suggest that non-histone crotonylation is associated with the occurrence and development of CRC. For example, Liao et al. reported that crotonic acid (CA) derived from the gut microbiome augments the proliferation of CRC cells by promoting crotonylation modification of P53-Ser46 [11]. Besides, Kcr of α enolase at K420 promotes the growth, migration, and invasion of CRC cells [12].

Crotonyl-CoA can release from mitochondria and then translocate into nuclei, where it triggers histone crotonylation. Xu et al. revealed that BRD4 promotes the migration and invasion of prostate cancer (PCa) cells through histone crotonylation [13]. However, at present, the effect of histone crotonylation on CRC progress has not been very clearly elucidated. Our previous findings revealed that the level of H3K27cr is significantly lowered during DNA damage in HCT116 cells [14]. Considering the strong association between DNA damage and CRC metastasis, H3K27cr could play a key role in CRC metastasis.

Modification of histone crotonylation is a reversible thermodynamic process regulated by crotonyltransferases and decrotonylases. SIRT3 is predominantly localized in the mitochondrial matrix and is generally thought to be a NAD-dependent deacetylase. Recently, Wei et al. showed that treating cells with NAM, a SIRT3 inhibitor, for 24 h increases histone crotonylation level [15]. Additionally, Bao et al. demonstrated that SIRT3 acts as a histone decrotonylase and regulates gene expression via altering histone lysine crotonylation. They further showed that SIRT3 recognizes and catalyzes the hydrolysis of crotonylated histone peptides [16]. Previous literature has shown that SIRT3 is also localized in nuclear and plays important roles in physiologic and pathophysiologic processes by regulating core histone modifications. However, the process by which SIRT3 is recruited to specific genomic loci is poorly understood.

Studies have revealed that long noncoding RNA (lncRNA) binds and recruits crotonylation-modifying enzymes to specific genome loci. For instance, lncRNA EPB41L4A-AS1 interacts with GCN5 and promotes its translocation to promoters of glucose metabolism-related genes [17]. Additionally, lncRNA NEAT1 interacts with P300/CBP complex, impacting the level of H3K27 crotonylation nearby promoters of endocytosis-related genes [18]. So far, the physical interaction between lncRNA and SIRT3 has not been reported yet.

LncRNA LINC00922 promotes the proliferation, migration, and invasion of colorectal, lung, gastric, ovarian, and liver cancer cells. Several studies have suggested that LINC00922 not only functions as a microRNA sponge, but also plays a role in promoting cancer metastasis by recruiting DNA methyltransferases (DNMTs) to the promoter regions of metastatic genes, resulting in altered expression of these genes [19, 20]. Our study found that H3K27cr and LINC00922 levels show a similar trend in CRC tissues and are higher in the distant metastatic CRC tissues than in primary tissues of CRC. Gene set enrichment analysis (GSEA) showed that LINC00922 level was associated with genes with promoter occupied by H3K27cr. Functionally, LINC00922 was involved in invasion and migration of CRC cells. Mechanistically, LINC00922 physically interacted with SIRT3 and prevented the translocation of SIRT3 to the ETS1 promoter, increasing the occupation of H3K27cr on the ETS1 promoter region and ETS1 expression. These events accelerated the invasion and migration of CRC cells both in vitro and in vivo. These discoveries suggested that H3K27cr functions as a crucial regulator of CRC metastasis.

## Materials and methods

### Cell culture

Human CRC cell lines, including HCT116 (TCHu 99), LoVo (TCHu 82), DLD-1 (TCHu134), SW620 (TCHu101), and SW480 (SCSP-5033), were purchased from the cell bank of the Chinese academy of science (Shanghai, China). Human normal colorectal epithelial cell line FHC (CRL-1831) was purchased from American type culture collection (Manassas, VA, USA). HCT116, LoVo, DLD-1, SW480, and FHC were cultured in Dulbecco’s modified eagle medium (DMEM) (Bio-Channel, Cat. No: BC-M-005) supplemented with 10% fetal bovine serum (FBS) (ExCell Bio, Cat. No: FSP500) and 1% antibiotics (Vicmed, Cat. No: VC2003). SW620 was cultured in RPMI 1640 medium (Bio-Channel, Cat. No: BC-M-017) supplemented with 10% FBS and 1% antibiotics. All cells were cultivated at 37 °C under 5% CO_2_ in a humidified incubator.

### Transfection and lentiviral transduction

The siRNAs were synthesized by integrated biotech solutions (Shanghai, China). The plasmids were purchased from Hebio (Shanghai, China) or YouBio (Hunan, China). The transfection of siRNAs and plasmids was performed in a transient manner, according to the manufacturer’s instructions, using siLentFect lipid reagent (Bio-Rad, Cat. No: 1,703,361) and liposomal transfection reagent (YEASEN, Cat. No: 40802ES08), respectively. The sh-LINC00922 plasmid was constructed and then packaged into lentivirus by GenePharma (Shanghai, China). To obtain LINC00922 stable knockdown cell line, HCT116 cells were infected with lentivirus and successfully transfected cells were screened using 4 µg/mL puromycin (Beyotime, Cat. No: ST551-10 mg) for two weeks. The sequences of siRNAs are shown in Table S1.

### Bioinformatics analysis

All the original clinical data and genomics information were downloaded from UCSC Xena (https://xena.ucsc.edu) and gene expression omnibus (GEO, https://www.ncbi.nlm.nih.gov/geo/) database. To compare the levels of LINC00922 and H3K27cr between two groups of samples, the Mann-Whitney U test was employed. The levels of H3K27cr and LINC00922 across multiple sample groups were analyzed using one-way analysis of variance (ANOVA) analysis. Kaplan-Meier analysis was performed to obtain survival analysis. A chi-square test was performed to analyze the distribution of LINC00922 between cytosol and nucleus. The expression correlation between LINC00922 and ETS1 in CRC tissues was examined using the Pearson correlation test. KEGG was used to investigate the biological pathways. Meta-analysis was performed to study the association between LINC00922 and CRC metastasis.

### Gene set enrichment analysis

GSEA was performed using the fgsea package (version 1.16.0) in R [21]. The CRC samples from the cancer genome atlas (TCGA) database were divided into two groups according to the median level of LINC00922. ChIP-seq data of H3K27cr and other crotonylation related gene sets downloaded from GEO database, were used in this analysis. The rank of genes was based on the sorting of fold-change and analyzed using the edgeR package (version 3.32.1) [22].

### Differential expression analysis

The lncRNAs differently expressed between normal and CRC tissues were analyzed using edgeR package. Briefly, gene expression profile was downloaded from UCSC Xena database. The samples were divided into two groups according to normal or cancer. The lncRNAs with |fold-change| ≥2 and *P* < 0.05 were displayed using heatmap.

### ScRNA-seq analysis

The single-cell transcriptomes of all samples from GSE196964 and GSE221575, four primary CRC tissues and two liver metastatic CRC tissues (GSM7058756 to GSM7058761) from GSE225857 were downloaded from GEO database. Following, data were analyzed using Seurat, presto, dplyr, msigdbr, tibble package in R. To remove the batch effects, the FindIntegrationAnchors function was employed to calculate the anchors, subsequently integrating all samples using the IntegrateData function. The marker of each cell-type was analyzed using FindAllMarkers function and then the cell-type was identified using CellMarker 2.0 database. The enrichment between gene sets and specific cell-type was analyzed using fgsea package and the results were displayed using ggplot2 package. The R code referred to the report of Wu et al. [23]. We also provided the R code of scRNA-seq data processing, including key parameters, in the supplementary material.

### Transwell assay

Cells were seeded into chambers containing 8 μm pores (Corning, Cat. No: 3422) with or without Matrigel (Corning, Cat. No: 356,234) coating. Briefly, 200 µL serum-free medium suspended with cells and 600 µL medium supplemented with 10% FBS were added to the upper and lower chambers in a bicameral culture system, respectively. After 24 and 48 h, cells penetrating the pores were fixed with 4% paraformaldehyde solution and stained with 0.1% crystal violet. Subsequently, cells were observed under an Olympus microscope at a magnification of × 100. Finally, the pictures captured were analyzed using Image J software.

### Wound healing assay

LoVo cells were seeded into a 6-well plate and cultured to 95% confluence. A 200 µL pipette tip was used to scratch on cell monolayers. Following the removal of cellular debris by washing, the cells in the plate were cultured with a new medium that was devoid of serum. Photomicrographs were obtained with a Nikon digital camera (Nikon, Toyko, Japan) at ×100 magnification at 0 and 48 h. Lastly, the change in the wound size was measured.

### Western blot

Total protein was harvested from cells using cell lysis buffer (50 mM Tris-HCl (pH 8.0), 4 M urea, and 1% Triton X-100) supplemented with protease inhibitors. To determine the concentration of cell lysates, the BCA kit (KeyGEN BioTECH, Cat. No: KGP902) was employed. Subsequently, proteins of varying molecular weights were separated through SDS-PAGE and transferred onto a nitrocellulose membrane. Subsequently, the membrane was blocked with 5% skim milk for 2 h and then incubated with primary antibodies overnight at 4 ℃, followed by secondary antibodies for 1 h. The following antibodies were used: anti-ETS1 (Beyotime, Cat. No: AF6812, 1:1,000), anti-E-Cad (ABclonal, Cat. No: A20798, 1:2,000), anti-Vimentin (Proteintech, Cat. No: 10366-1-AP, 1:2,000), anti-Snail1 (Proteintech, Cat. No: 13099-1-AP, 1:1,000), anti-SIRT3 (Santa Cruz, Cat. No: sc-365,175, 1:200), anti-H3K27cr (PTM BIO, Cat. No: PTM-545RM, 1: 1,000), anti-GAPDH (Proteintech, Cat. No: 60004-1-Ig, 1:10,000), anti-H3 (Proteintech, Cat. No: 17168-1-AP, 1:10,000), anti-rabbit IgG (SAB, Cat. No: L3012, 1:10,000), and anti-mouse IgG (SAB, Cat. No: L3032, 1:10,000). The intensity of bands was scanned and then measured using Image J software.

### Quantitative real-time PCR

Total RNA from tissues and cells was extracted using TRIzol reagent. The RNA was then reverse transcribed into cDNA using SweScript RT I First Strand cDNA Synthesis Kit (Servicebio, Cat. No: G3330-50). QRT-PCR was performed using 2×SYBR Green qPCR Master Mix (High ROX) (Servicebio, Cat. No: G3322-05). The qPCR process was performed in triplicate, and the relative fold-change of genes was normalized using GAPDH and calculated using the 2^−ΔΔCt^ formulae. Primers used for qPCR are listed in Table S2.

### Immunofluorescence staining

The immunofluorescence assay was conducted using antibodies against ETS1 (1:50), H3K27cr (1:100), and SIRT3 (1:50). The experiment began with the seeding of cells onto coverslips, followed by a 24 h growth period. Afterwards, the cells were washed three times with PBS and fixed with cold methanol for 5 min. Then, the samples were blocked with 3% bovine serum albumin (BSA) for 1 h and incubated with primary antibody for 2 h at room temperature. Subsequently, the glasses were immersed into 3% FBS containing Alexa Fluor 594-conjugated Goat Anti-Rabbit IgG(H + L) (Vicmed, Cat. No: VA027, 1:100) and/or Alexa Fluor 488-conjugated Affinipure Goat Anti-Mouse IgG(H + L) (Vicmed, Cat. No: VA1021, 1:100) for 1 h at room temperature. Finally, the cells were stained with DAPI and visualized using a fluorescence microscope or STELLARIS 5 confocal fluorescence microscope (Leica).

### Chromatin immunoprecipitation (ChIP-seq and ChIP-qPCR) assays

Cells were cross-linked with 1% formaldehyde and then re-suspended in ChIP lysis buffer (50 mM Tris-HCl (PH = 8.0), 5 mM EDTA, 0.1% deoxycholate, 1% Triton X-100, 150 mM NaCl and 20 µL/mL PIC). After 10 min, cells were sonicated and centrifuged. The supernatants were subsequently incubated with antibodies and Protein A/G beads for 4 h. In the ChIP assay, anti-H3K27cr and anti-SIRT3 antibodies were employed, while rabbit IgG (Beyotime, Cat. No: A7016) and mouse IgG (Bioworld, Cat. No: BD0050) served as a control. This was followed by washing of the complex samples thrice to obtain clean DNA samples. Finally, DNA samples were analyzed using qRT-PCR assay or sequenced by Orizymes company (Shanghai, China).

### Cell fractionation assay

RNA in the cytosol and nucleus was extracted using nuclear and cytoplasmic protein extraction kit (Beyotime, Cat. No: P0027). Firstly, PMSF and RRI were added into cytoplasmic protein extraction reagent A. Then, cells were lysed with pre-cooling reagents A and B on ice for 15 min. Subsequently, cell lysate was centrifuged, and the supernatant was collected to extract cytoplasmic RNA. The sediment was resuspended in reagent A and reagent B while kept on ice for 45 min, and subsequently washed thrice with PBS to remove any remaining cytoplasm. Finally, Trizol was used to extract nuclear RNA from the centrifuged deposit.

### RNA immunoprecipitation assay

Cells were harvested and then digested using RIP lysis buffer (10 mM KCl, 5 mM MgCl_2_, 10 mM HEPES (PH = 7.0), 0.5% NP-40, 1 mM DTT, 100 U/mL RRI, 20 µL/mL protease inhibitor and 2 mM vanadyl ribonucleotide complex solution). Subsequently, the cell lysate was mixed with 3 µg of IP-grade anti-SIRT3 and left to incubate overnight at 4 °C, and mouse IgG antibodies served as a control. The reaction mixture was then supplemented with 40 µL of Protein A/G beads and left to react for 4 h. The resulting precipitated RNA was extracted, purified and subjected to qRT-PCR analysis.

### Fluorescent in situ hybridization (RNA-FISH) and immunofluorescence microscopy

The LINC00922 RNA probe labeled with Cyanine 5 was bought from integrated biotech solutions (Shanghai, China). Briefly, the cells were spread on coverslips in a 12-well plate. The following day, the coverslips were washed three times with PBS, and the cells were permeabilized with CSK buffer on ice for 5 min and then fixed with 4% paraformaldehyde for 10 min. Then, cells were successively dehydrated with 80, 95, and 100% ethanol for 3 min at each concentration and incubated with the LINC00922 probe at 37 °C overnight in a dark and humid chamber. The coverslips were then washed three times in freshly-prepared 50% formamide and 2 × SSC buffer at 42 °C for 5 min. Subsequently, the cells were blocked with 1% BSA for 15 min and then exposed to SIRT3 antibody (1:50) for 45 min. Finally, the specimens were counterstained with DAPI and mounted to be examined on a STELLARIS 5 confocal fluorescence microscope (Leica).

### Immunohistochemistry (IHC)

Tumor tissues were purchased from Shanghai Outdo Biotech company (Shanghai, China), including colorectal tumor samples from 45 patients and adjacent tissues from 14 patients. The 45 tumor samples in contained 10 distant metastatic colorectal cancer tissues, 16 primary colorectal cancer tissues, 11 colorectal adenoma tissues, and 8 positive lymph node tissues. Tissues were incubated twice in xylene for 15 min each time and then in 100% ethanol twice for 7 min each time, and once in 90, 80, and 70% ethanol for 5 min each. Afterward, 0.01 M citrate buffer was used for antigen retrieval and then 3% hydrogen peroxide was used to quench peroxidase activity. Tissues were blocked with BSA, then incubated with anti-H3K27cr antibody (1:100) at 4 °C overnight, and then incubated with the secondary antibody labeled with HRP (SAB, Cat. No: L3012, 1:100) for 30 min at room temperature. Next, the slices were dehydrated in succession, sealed with neutral resin, and scrutinized under a microscope. Ultimately, slices were evaluated independently and blindly by two observers.

**Fig. 1:**
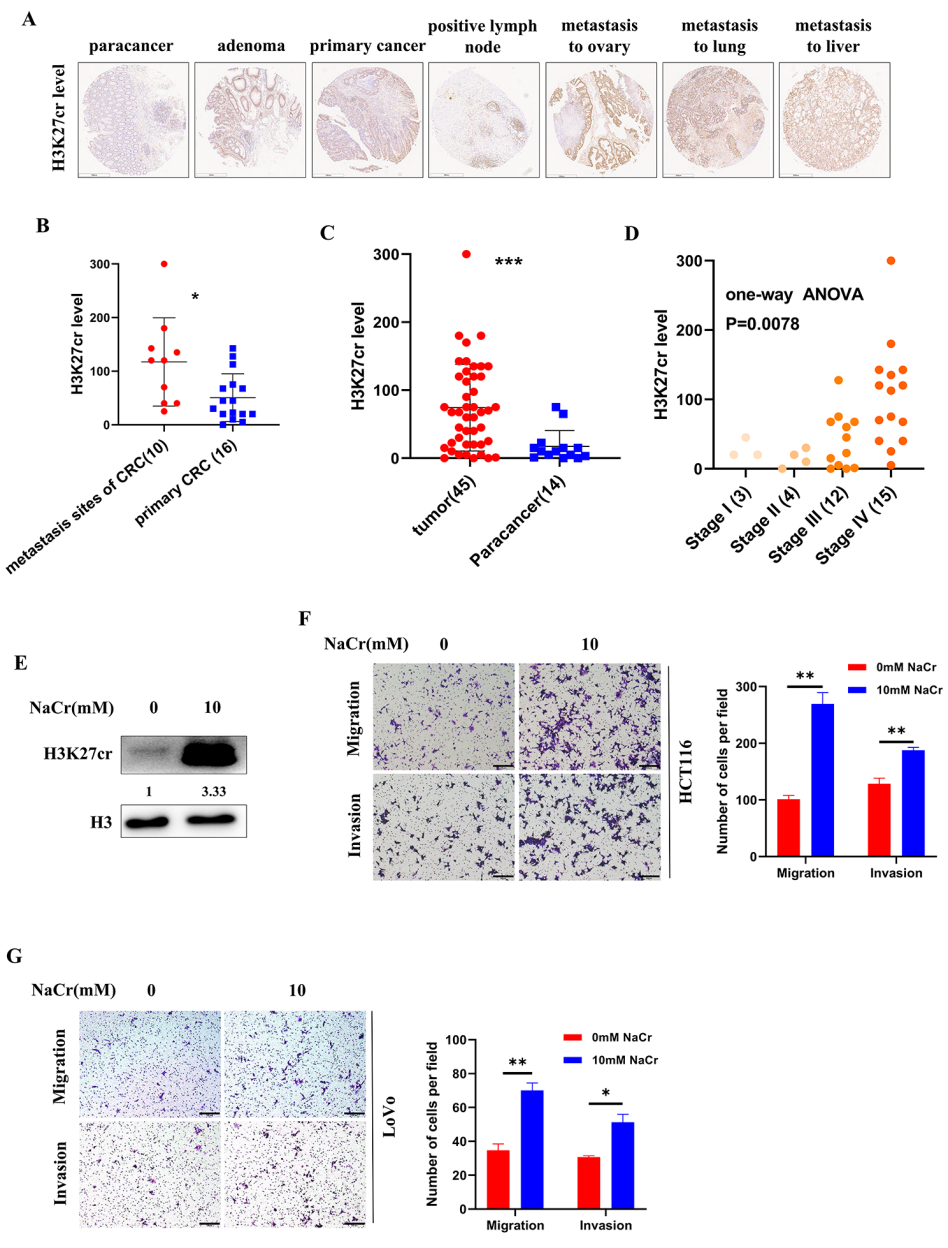
Clinical results showing H3K27cr was associated with CRC metastasis. (**A**) Representative immunohistochemistry images depicting H3K27cr level across different types of CRC tissues. Scale bar, 500 μm. (**B-D**) Mann-Whitney U (B-C) and one-way ANOVA (D) analysis of H3K27cr level across different types of CRC tissues. The 45 cases of tumor samples in Fig. 1C contained 10 cases of distant metastatic colorectal cancer tissues, 16 cases of primary colorectal cancer tissues, 11 cases of colorectal adenoma tissues, and 8 cases positive lymph node tissues. (**E**) Immunoblotting analysis of H3K27cr level in HCT116 cells treated with 10 mM NaCr for 48 h. The numbers below H3K27cr indicate the ratio of H3K27cr versus H3 in experimental groups to that in control group, analyzed using Image J2. (**F-G**) Transwell assay images demonstrating the levels of invasion and migration in HCT116 (F) and LoVo cells (G) treated with 10 mM NaCr for 48 h (left panel). Scale bar, 200 μm. Cells were counted in 3 random fields (right panel). Data are represented as means ± SD, *P < 0.05, **P < 0.01, ***P < 0.001, unpaired, two-tailed, Student’s *t*-test

The levels of H3K27cr were quantified using histochemistry score (H-score), with multiplication of the staining intensity with the positive cell area. Staining intensity was evaluated using a grade system ranging from 0 to 3, where 0 indicating no staining, 1 indicating weak staining, 2 indicating moderate staining, and 3 indicating strong staining. The positive cell area was measured as a percentage ranging from 0 to 100%. As a result, the H3K27cr levels spanned from 0 to 300%. In Fig. 1B and D, a comparative analysis of the continuous levels of H3K27cr was conducted across multiple groups. In Tables 1 and 2, the positive cell area was converted into a grading system ranging from 0 to 4: 0 (< 5%), 1 (5-25%), 2 (25-50%), 3 (50-75%), 4 (> 75%). Multiplying two grades resulted in a score ranging from 0 to 12, with H3K27cr scores larger than 6 considered as high levels. Four-table chi-square test was applied to analyze the significance.

**Table 1:** Correlation between H3K27cr level and clinicopathological characteristics

	variables	H3K27cr level		total	Chi-square value	*p*-value
		low	high			
Sex					0.56	0.45
	male	20	6	26		
	Female	12	6	18		
	NA			1		
Age (year)					0.007	0.93
	≤ 54	14	4	18		
	> 54	15	4	19		
	NA			8		
Grade					5.08	0.024
	G1-3	14	4	18		
	G4	3	6	9		
	NA			18		
Tumor size					0.17	0.68
	≤ 5 cm	16	3	19		
	> 5 cm	11	3	14		
	NA			12		
T stage					0.71	0.40
	T2	3	0	3		
	T3	8	2	10		
	NA			32		
N stage					0.90	0.34
	N0	7	0	7		
	N1/N2	15	2	17		
	NA			21		
M stage					7.99	0.005
	M0	18	1	19		
	M1	8	7	15		
	NA			11		
TNM stage					7.99	0.005
	I-III	18	1	19		
	IV	8	7	15		
	NA			11		

**Table 2:** Differential level of H3K27cr in cancer and paracancer

	H3K27cr level		n	Chi-square value	*p*-value
	low	high			
Cancer	32	13	45	5.19	0.023
Paracancer	14	0	14		

### Animal experiment

To explore the effect of LINC00922 in the CRC metastasis, nude mice were used to construct a lung metastasis model. The BALB/c nude mice were developed at GemPharmatech (Nanjing, China) and were randomly divided into two groups (n = 8/group). A sum of 3 × 10^6^ HCT116 cells, with or without stable LINC00922 knockdown, were injected into each mouse via the tail vein. Four mice injected with control cells died during animal experiment. After five weeks, four and eight mice in the control and sh-LINC00922 groups were killed, respectively. The lungs of mice with tumor implant nodules were collected, photographed, and statistically analyzed.

### Statistical analysis

All experiments were performed in independent triplicates, and the data were presented as mean ± SD. Statistical analysis was performed using SPSS software version 22.0, and graphs were plotted using GraphPad Prism software version 8.0. Differences between the two groups were evaluated using an unpaired two-tailed Student’s *t*-test, Mann-Whitney U test, or Chi-square test. Differences among several groups were evaluated using one-way ANOVA. Association between the two groups was evaluated using Pearson’s correlation analyses. *P* < 0.05 was considered statistically significant.

## Results

### Clinically, H3K27cr level is associated with metastasis of CRC tissues

We conducted an investigation into the level of H3K27cr in colorectal tumor samples obtained from 45 patients, as well as adjacent tissues from 14 patients, utilizing immunohistochemical staining. Intriguingly, the findings demonstrated a significant elevation in H3K27cr level at distant metastasis sites (Fig. 1A-B). Furthermore, a correlation between H3K27cr level and the grade, M stage, and TNM stage of CRC tissues was observed (Table 1). Notably, when compared to Para-cancer tissues, tumor tissues exhibited a significantly higher H3K27cr level (Fig. 1C; Table 2). Besides, the H3K27cr level was higher in stage III and stage IV tissues than in stage I and stage II (Fig. 1D). Given the concomitant relationship between distant metastasis and H3K27cr level, we put forth the hypothesis that H3K27cr could potentially enhance cancer metastasis. NaCr increased histone crotonylation level by directly producing crotonyl-CoA [24]. To test our hypothesis, the HCT116 cells were cultured in a medium supplemented with 10 mM NaCr to improve the intracellular level of H3K27cr (Fig. 1E). The results showed that NaCr promoted the invasion and migration of CRC cells (Fig. 1F-G). These findings suggested that H3K27cr promoted CRC metastasis.

### LINC00922 is associated with H3K27cr level, metastasis and poor prognosis in CRC

**Fig. 2:**
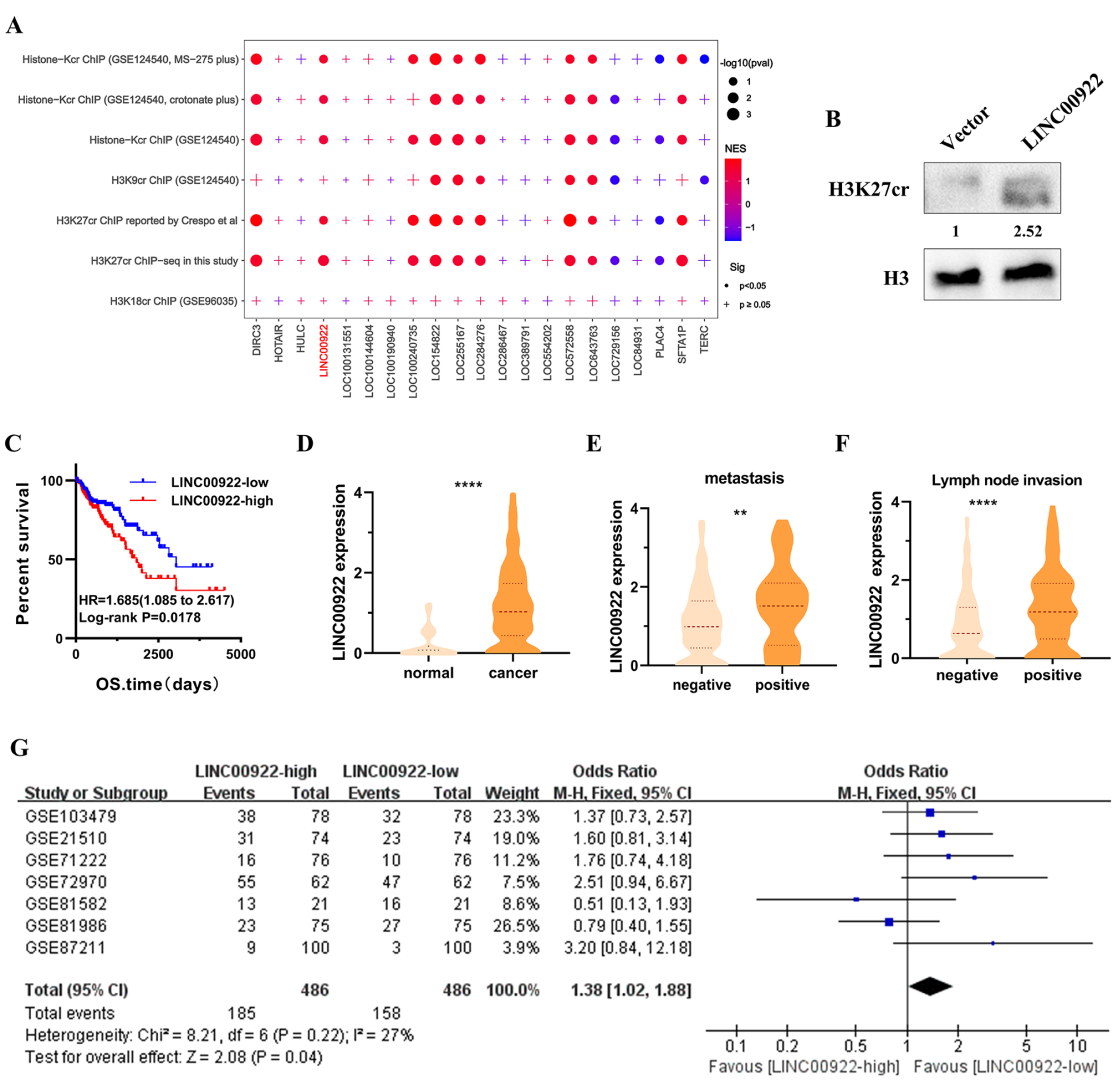
Association of LINC00922 with CRC metastasis and poor prognosis. (**A**) Enrichment of genes with promoter occupied by histone crotonalytion across various dysregulated lncRNAs. The significance was calculated using GSEA. Dot indicates *P* < 0.05, and plus symbol indicates *P* ≥ 0.05. The red point represents gene set positively expressed with the corresponding lncRNA, and the blue point represents the opposite. The size of point represents the negative base 10 logarithm of *p*-value. NES: normalized enrichment score. (**B**) Immunoblotting analysis of H3K27cr level in HCT116 cells transfected with LINC00922 plasmid for 48 h. The numbers below H3K27cr indicate the ratio of H3K27cr versus H3 in experimental groups to that in control group, analyzed using Image J2. (**C**) Kaplan-Meier survival curve analysis of the association of LINC00922 expression with overall survival (OS, n = 326) in CRC samples from the TCGA cohort. (**D**) The Mann-Whitney U analysis of LINC00922 expression level between normal and CRC tissues from the TCGA database. ****P < 0.0001. **E-F.** Mann-Whitney U analysis of LINC00922 expression in CRC samples from the TCGA cohort with or without distant metastasis (E), or with or without lymph node invasion (F). **P < 0.01, ****P < 0.0001. **G.** Meta-analysis of the relationship between LINC00922 expression and distant metastasis in CRC samples (n = 972) from the GEO database

Since previous studies indicate that lncRNA plays essential roles in regulating histone crotonalytion [17], the association between H3K27cr and lncRNAs was next studied. Firstly, an analysis was conducted to identify lncRNAs that exhibited differential expression in normal and CRC tissues from the TCGA cohort, revealing dysregulation of multiple lncRNAs in CRC tissues (Fig. S1A). Subsequently, ChIP-seq assay was performed in HCT116 cells utilizing H3K27cr antibody, and genes with promoter occupied by H3K27cr were extracted (Table S3). Afterwards, GSEA was performed to compare these genes with the aforementioned dysregulated lncRNAs. The result discovered that these genes were enriched in CRC tissues exhibiting high expression of LINC00922 or eight other lncRNAs (Fig. 2A). Conversely, no significant relationship was observed between LINC00922 and genes with promoters marked by H3K9cr or H3K18cr. Notably, overexpression of LINC00922 significantly elevated the level of H3K27cr (Fig. 2B). These results suggested a correlation between LINC00922 and H3K27cr.

Subsequently, an investigation into the clinical significance of LINC00922 was undertaken in CRC patients from the TCGA cohort. The results revealed that high expression of LINC00922 was associated with shorter survival of CRC patients (Fig. 2C). We next examined the correlation between LINC00922 and the clinicopathological characteristics of CRC tissues from the TCGA cohort. The Mann-Whitney U analysis revealed that LINC00922 expression was significantly elevated in CRC tissues compared to normal tissues (Fig. 2D). Furthermore, the level of LINC00922 was higher in several types of CRC cells than in the normal colorectal epithelial cells (Fig. S1B). Besides, the expression of LINC00922 was higher in CRC patients with distant or lymph node metastasis than those without distant or lymph node metastasis (Fig. 2E-F). Additionally, a meta-analysis of GEO cohort revealed that high expression of LINC00922 increased risk of distant metastasis (odds ratio (OR) = 1.38 (95% CI: 1.02 ~ 1.88, Z = 2.08, *P* = 0.04)) (Fig. 2G). In summary, the results suggested that high level of LINC00922 increased the risk of CRC metastasis and was associated with poor prognosis of the CRC patients.

### LINC00922 accelerates invasion and migration of CRC cells

**Fig. 3:**
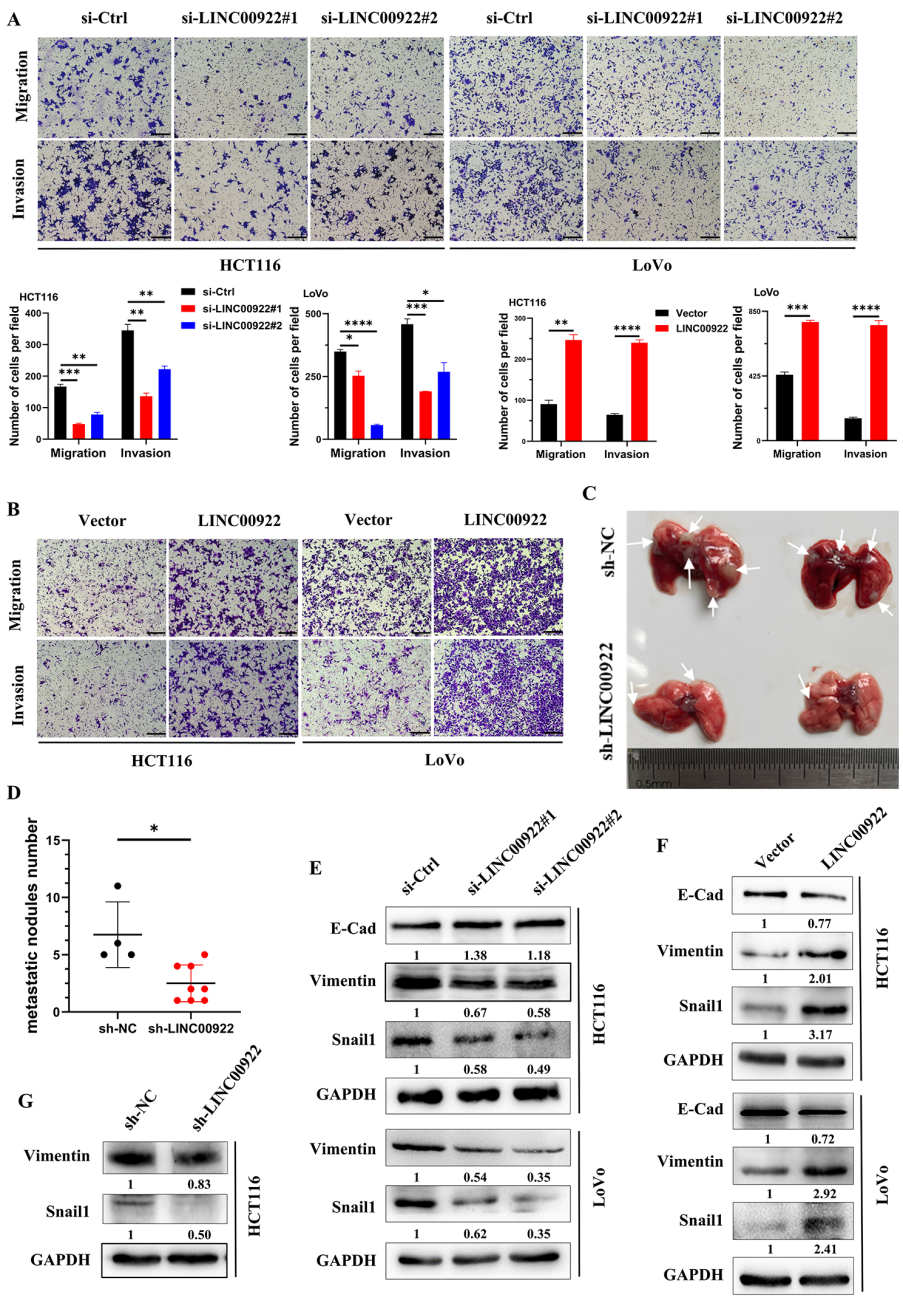
LINC00922 promoted CRC metastasis. (**A-B**) Representative images of transwell assay showing the invasion and migration levels of CRC cells after instantaneous knockdown (A) or overexpression (B) of LINC00922 for 48 h. Scale bar, 200 μm. Cells were counted in 3 random fields (middle panel). (**C**) Illustrations of LINC00922 stable knockdown HCT116 cell-derived lung metastases. The arrow indicates the tumor nodule. (**D**) Comparison of lung metastasis nodules between the sh-NC and sh-LINC00922 groups. (**E-G**) Immunoblotting analysis of E-Cad, Vimentin, Snail1, and GAPDH levels in CRC cells with LINC00922 transient knockdown (E), transient overexpression (F), or stable knockdown (G). The numbers below western bands indicate the ratio of protein versus GAPDH in experimental groups to that in control group, analyzed using Image J2. Data are represented as means ± SD, *P < 0.05, **P < 0.01, ***P < 0.001, ****P < 0.0001, unpaired, two-tailed, Student’s *t*-test

To further explore the relationship between LINC00922 and CRC metastasis, we investigated the role of LINC00922 on the invasion and migration of CRC cells. The results showed that depletion of LINC00922 decreased the level of invasion and migration of CRC cells, whereas overexpression of LINC00922 revealed the opposite effect (Fig. 3A-B, and Fig. S2A-B), consistent with the result of wound healing assay (Fig. S2C-D). Whether LINC00922 regulated tumor metastasis in vivo was also investigated. Stable LINC00922 knockdown cells were injected into mice via the tail vein. The results revealed that LINC00922 knockdown significantly reduced the metastatic nodules in lung (Fig. 3C, Fig. S2E). During the animal experiments, four mice injected with sh-NC cells succumbed to mortality. Ultimately, a cumulative count of 27 metastatic tumor nodules was obtained from the four mice injected with sh-NC cells, resulting in an average of 6.75 nodules per mouse. A total of 20 metastatic tumor nodules were collected from eight mice injected with sh-LINC00922 cells, yielding an average of 2.5 nodules per mouse (Fig. 3D). Immunoblotting analysis demonstrated that the expression levels of EMT-related genes, such as Vimentin and Snail1, were significantly downregulated upon knockdown of LINC00922 in CRC cells. Conversely, overexpression of LINC00922 had an opposite effect (Fig. 3E-G), increasing the expression levels of these genes. These findings collectively suggested that LINC00922 promoted invasion and migration of CRC cells.

### LINC00922 promotes invasion and migration via H3K27cr mediated CAMs in epithelial cells

**Fig. 4:**
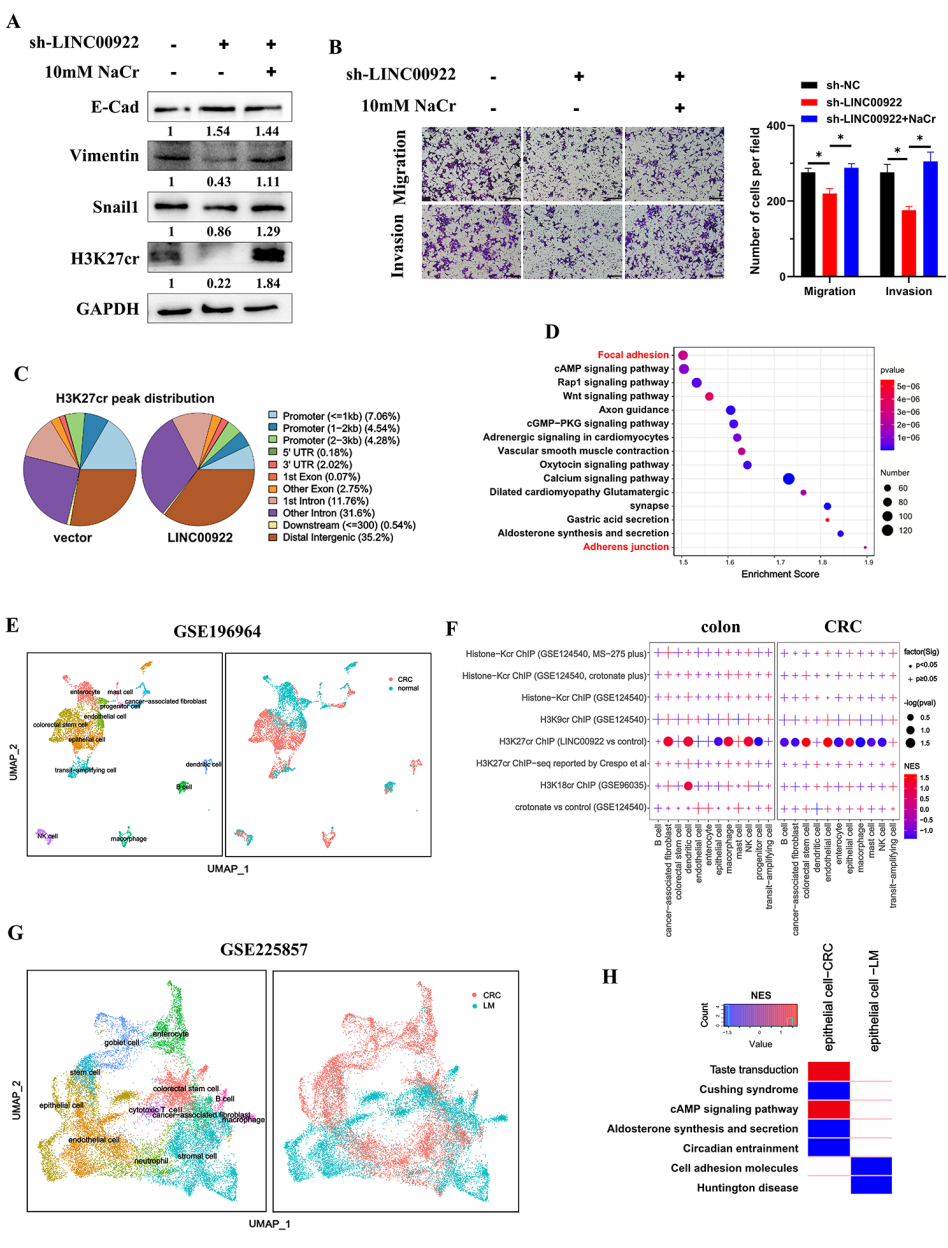
LINC00922 promoted invasion and migration via H3K27cr mediated CAMs in epithelial cells. (**A**) Immunoblotting analysis of E-Cad, Vimentin, and Snail1 expression in LINC00922 stable knockdown HCT116 cells treated with 10 mM NaCr for 48 h. The numbers below western bands indicate the ratio of protein versus GAPDH in experimental groups to that in control group, analyzed using Image J2. (**B**) Transwell assay images demonstrating the level of invasion and migration in LINC00922 stable knockdown HCT116 cells treated with 10 mM NaCr for 48 h (left panel). Scale bar, 200 μm. Cells were counted in 3 random fields (right panel). Data are represented as means ± SD, *P < 0.05, unpaired, two-tailed, Student’s *t*-test. (**C**) H3K27cr peaks distribution in HCT116 cells transfected with the LINC00922 or control plasmid for 48 h. (**D**) KEGG analysis of enrichment pathways of genes annotated by 21,253 different H3K27cr peaks in HCT116 cells with or without transient LINC00922 overexpression for 48 h. (**E**) UMAP plotting single cell transcriptome profiles of CRC tissues (GSE196964). The color represents different cell types (left panel) or tissue types (right panel). (**F**) Enrichment of 653 genes and other histone crotonylation-related gene sets across various cell types of colon tissues (left panel) and CRC tissues (right panel). The significance was calculated using GSEA. Dot indicates *P* < 0.05, and plus symbol indicates *P* ≥ 0.05. The red point represents gene set positively expressed in the corresponding cell type, and the blue point represents the opposite. The size of dot represents the negative base 10 logarithm of *p*-value. (**G**) UMAP plotting single cell transcriptome profile of CRC tissues (GSE225857). The color represents different cell types (left panel) or tissue types (right panel). (**H**) Heatmap showing the enrichment of pathways annotated by 653 genes in epithelial cells derived from primary CRC and LM tissues. The NES was calculated using GSEA. The red represents pathway positively enriched in the epithelial cells, and the blue represents the opposite. LM: liver metastasis

To investigate whether LINC00922 regulated invasion and migration of CRC cells via H3K27cr, LINC00922 stable knockdown cells were treated with 10 mM NaCr for 48 h to restore H3K27cr level. The results showed that NaCr supplementation restored the levels of E-cad, Vimentin and Snail1, and the invasion and migration of stable LINC00922 knockdown cells (Fig. 4A-B), suggesting that LINC00922 regulated invasion and migration of CRC cells via H3K27cr. Subsequently, the ChIP-seq assay was conducted in HCT116 cells overexpressing LINC00922 using H3K27cr antibody. A total of 21,253 peaks exhibited differential occupancy (|fold-change| > 1.2, *P* < 0.05) between LINC00922 overexpressed and control cells (Fig. 4C, Table S4), suggesting that LINC00922 influenced the occupation of H3K27cr on chromosomes. KEGG analyzing the biological pathways of genes annotated by above 21,253 peaks found an enrichment of metastatic pathways, including focal adhesion and adherens junction (Fig. 4D). For previous studies show that histone crotonylation robustly indicates active promoters, we subsequently extracted 653 genes with two peaks within promoter region for further study (Table S5).

To identify the specific cell types regulated by LINC00922 via H3K27cr, enrichment of these 653 genes in single-cell transcriptomes of CRC tissues was analyzed using GSEA. This analysis involved a total of 5678 cells from the GSE196964 dataset, leading to the identification of 12 major cell subtypes (Fig. 4E, Fig. S3A). In colon tissues, the 653 genes showed enrichment in dendritic cells, cancer-associated fibroblasts, NK cells, macrophages, progenitor cells, and epithelial cells. In CRC tissues, the 653 genes were enriched in all cell types, except for dendritic cells and transit-amplifying cells (Fig. 4F). The same analysis was performed across other dataset. A total of 5179 cells from the GSE221575 dataset were analyzed, leading to the identification of 11 major cell subtypes (Fig. S3B-C). In colon tissues, a predominant enrichment of the 653 genes in endothelial and epithelial cells was observed. In CRC and liver metastasis (LM) CRC tissues, a predominantly enrichment in B cell and epithelial cells was observed (Fig. S3D). Therefore, epithelial cells were next focused.

To investigate the regulatory pathways influenced by LINC00922 via H3K27cr, we annotated the biological pathways associated with these 653 genes. Our findings revealed that 79 pathways, such as focal adhesion, adherens junction, tight junction, and cell adhesion molecules (CAMs), contained more than 5 genes (Fig. S3E, Table S5). In the GSE225857 dataset, which encompassed CRC and LM tissues, a comprehensive analysis of 20,753 cells was performed, resulting in the identification of 12 major cell subtypes (Fig. 4G). The 79 pathways were compared to the epithelial cells extracted from CRC and LM tissues, respectively. The result found that pathway of CAMs was negatively enriched in epithelial cells derived from LM tissues, but not in those from CRC tissues (Fig. 4H). Consequently, these findings suggested that LINC00922 promoted invasion and migration through the H3K27cr-mediated expression of CAMs in epithelial cells of CRC tissues.

### LINC00922 promotes invasion and migration via ETS1

**Fig. 5:**
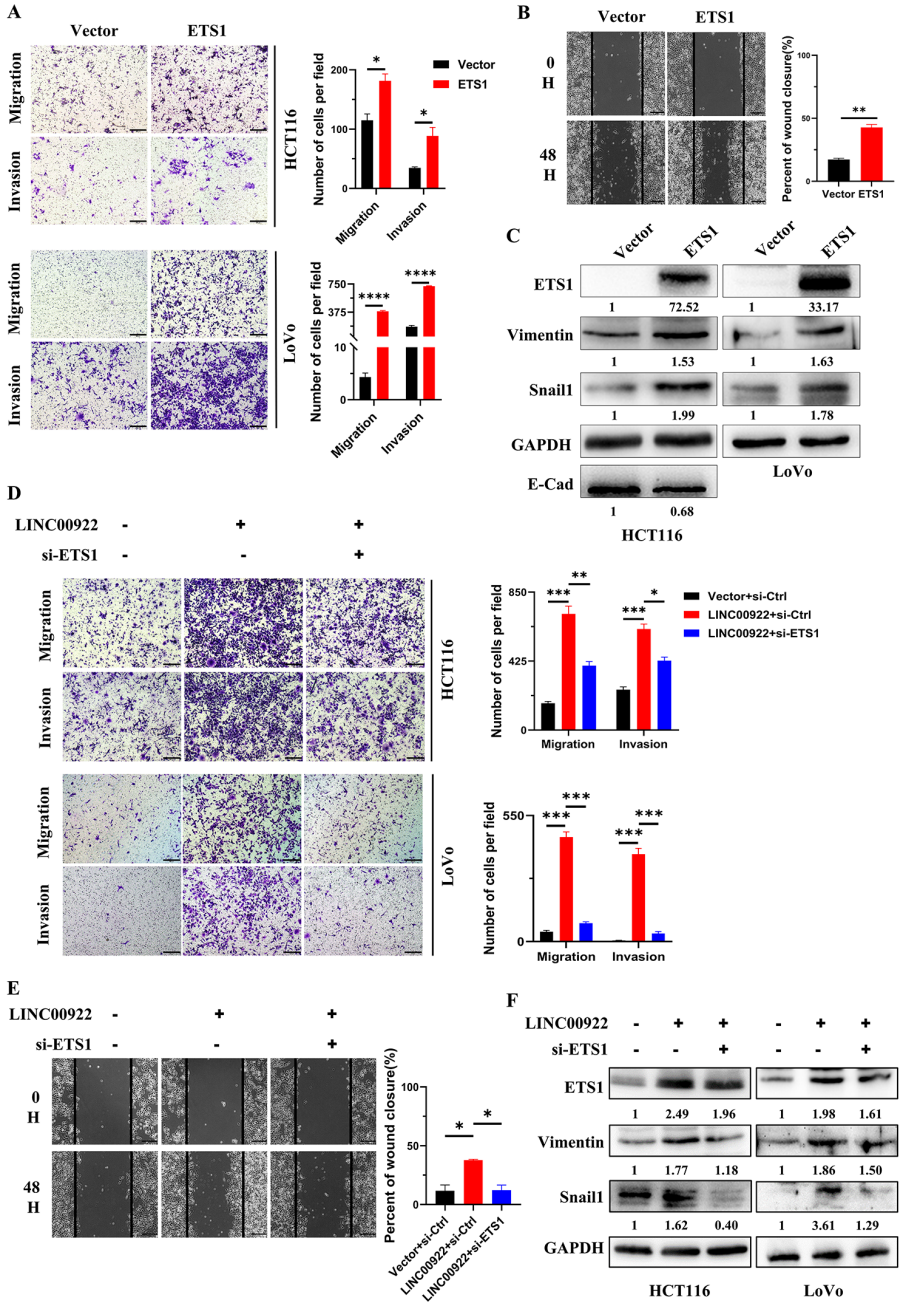
LINC00922 promoted invasion and migration via ETS1. (**A-B**) Representative images of Transwell (A) and wound healing assay (B) depicting motility of cells following ETS1 overexpression for 48 h (left panel). Scale bar, 200 μm. The number of cells and percent of wound closure were counted in 3 random fields (right panel). (**C**) Immunoblotting analysis of E-Cad, Vimentin, Snail1, ETS1, and GAPDH levels in CRC cells subjected to ETS1 transient overexpression for 48 h. (**D-E**) Representative images of Transwell assay (D) and wound healing assay (E) depicting motility in HCT116 cells co-transfected with LINC00922 plasmid and si-ETS1 for 48 h (left panel). Scale bar, 200 μm. The number of cells and percent of wound closure were counted in 3 random fields (right panel). (**F**) Immunoblotting analysis of Vimentin, Snail1, ETS1, and GAPDH levels in CRC cells co-transfected with LINC00922 plasmid and si-ETS1 for 48 h. The numbers below western bands indicate the ratio of protein versus GAPDH in experimental groups to that in control group, analyzed using Image J2. Data are represented as means ± SD, *P < 0.05, **P < 0.01, ***P < 0.001, ****P < 0.0001, unpaired, two-tailed, Student’s *t*-test

Previous studies indicate that ETS1 regulates the expression of CAMs. Therefore, we next investigated whether LINC00922 regulated invasion and migration via ETS1. Firstly, this study demonstrated that exogenous ETS1 promoted the invasion, migration, and cell motility of CRC cells as well (Fig. 5A-B). Additionally, overexpression of ETS1 increased the expression of Vimentin and Snail1, and decreased the expression of E-cad (Fig. 5C). The rescue assay demonstrated that inhibition of ETS1 restored the levels of invasion, migration, and cell motility in CRC cells by restoring Vimentin and Snail1 levels (Fig. 5D-F). These findings suggested that LINC00922 modulated invasion and migration through ETS1.

### LINC00922 regulates ETS1 expression by modulating H3K27cr level

**Fig. 6:**
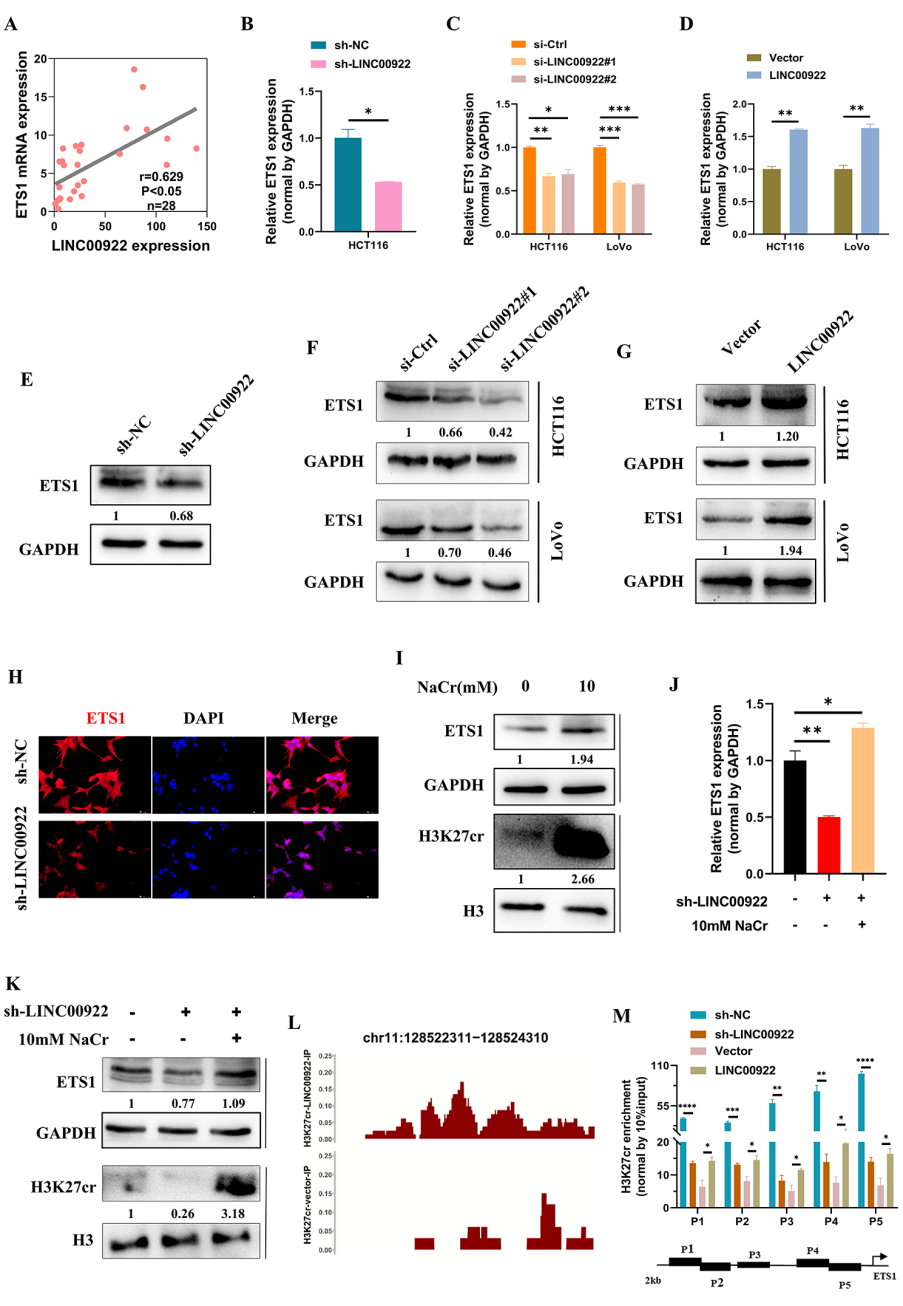
LINC00922 regulated ETS1 expression via H3K27cr. (**A**) qRT-PCR analysis of association between LINC00922 and ETS1 expression in CRC tissues (n = 28). (**B-G**) qRT-PCR analysis (B-D) and immunoblotting analysis (E-G) of ETS1 expression in HCT116 cells with LINC00922 stable knockdown (B, E), or CRC cells transfected with si-LINC00922 (C, F) or LINC00922 plasmid (D, G) for 48 h. (**H**) Immunofluorescence images of ETS1 (red) in HCT116 cells with LINC00922 stable knockdown. Scale bar, 100 μm. (**I**) Immunoblotting analysis of ETS1, H3K27cr, H3, and GAPDH levels in HCT116 cells treated with 10 mM NaCr for 48 h. (**J-K**) qRT-PCR (J) and immunoblotting (K) analysis of ETS1 expression in LINC00922 stable knockdown HCT116 cells supplemented with 10 mM NaCr for 48 h. (**L**) Image showing H3K27cr enrichment on the EST1 promoter region in HCT116 with LINC00922 transient overexpression for 48 h. (**M**) ChIP-qPCR assay analysis of H3K27cr enrichment on the EST1 promoter in HCT116 cells treated with LINC00922 stable knockdown or transient overexpression for 48 h. The numbers below western bands indicate the ratio of protein versus GAPDH or H3 in experimental groups to that in control group, analyzed using Image J2. Data are represented as means ± SD, *P < 0.05, **P < 0.01, ***P < 0.001, ****P < 0.0001, unpaired, two-tailed, Student’s *t*-test

Next, we investigated whether LINC00922 altered ETS1 expression. We conducted the qRT-PCR analysis in CRC tissues from 28 patients. The result exhibited a direct correlation between the expression of LINC00922 and ETS1 (Fig. 6A). Knockdown of LINC00922 expression revealed that both instantaneous and stable knockdown of LINC00922 reduced the ETS1 mRNA level (Fig. 6B-C). Contrarily, overexpression of LINC00922 in CRC cells increased the mRNA level of ETS1 (Fig. 6D). Moreover, the protein level of ETS1 showed a similar tendency (Fig. 6E-G). The fluorescent images also revealed that stable LINC00922 knockdown significantly reduced the protein level of ETS1 (Fig. 6H).

Whether LINC00922 regulated ETS1 expression via H3K27cr was investigated. Firstly, HCT116 cells treated with 10 mM NaCr increased the levels of H3K27cr and ETS1, indicating H3K27cr might activate ETS1 transcription (Fig. 6I). Afterward, 10 mM NaCr was added to the culture media of stable LINC00922 knockdown cells, followed by 48 h incubation to restore the H3K27cr level. We found that NaCr restored ETS1 expression of LINC00922 stable knockdown cells, suggesting that LINC00922 regulated ETS1 expression by modulating H3K27cr (Fig. 6J-K). Analysis of LINC00922 distribution between the nucleus and cytoplasm revealed that most LINC00922 RNAs were concentrated in the nucleus (Fig. S4). The ChIP-seq data showed that LINC00922 overexpressed increased the occupation of H3K27cr on promoter region of ETS1 (Fig. 6L). This result was confirmed by ChIP-qPCR as well, described as silencing LINC00922 significantly decreased the occupation of H3K27cr on the promoter region of ETS1, whereas exogenous LINC00922 exerted an opposite effect (Fig. 6M). In conclusion, our findings suggested that LINC00922 altered the enrichment level of H3K27cr on the ETS1 promoter, leading to a change in ETS1 expression.

### LINC00922 alters H3K27cr occupation by interacting with SIRT3

**Fig. 7:**
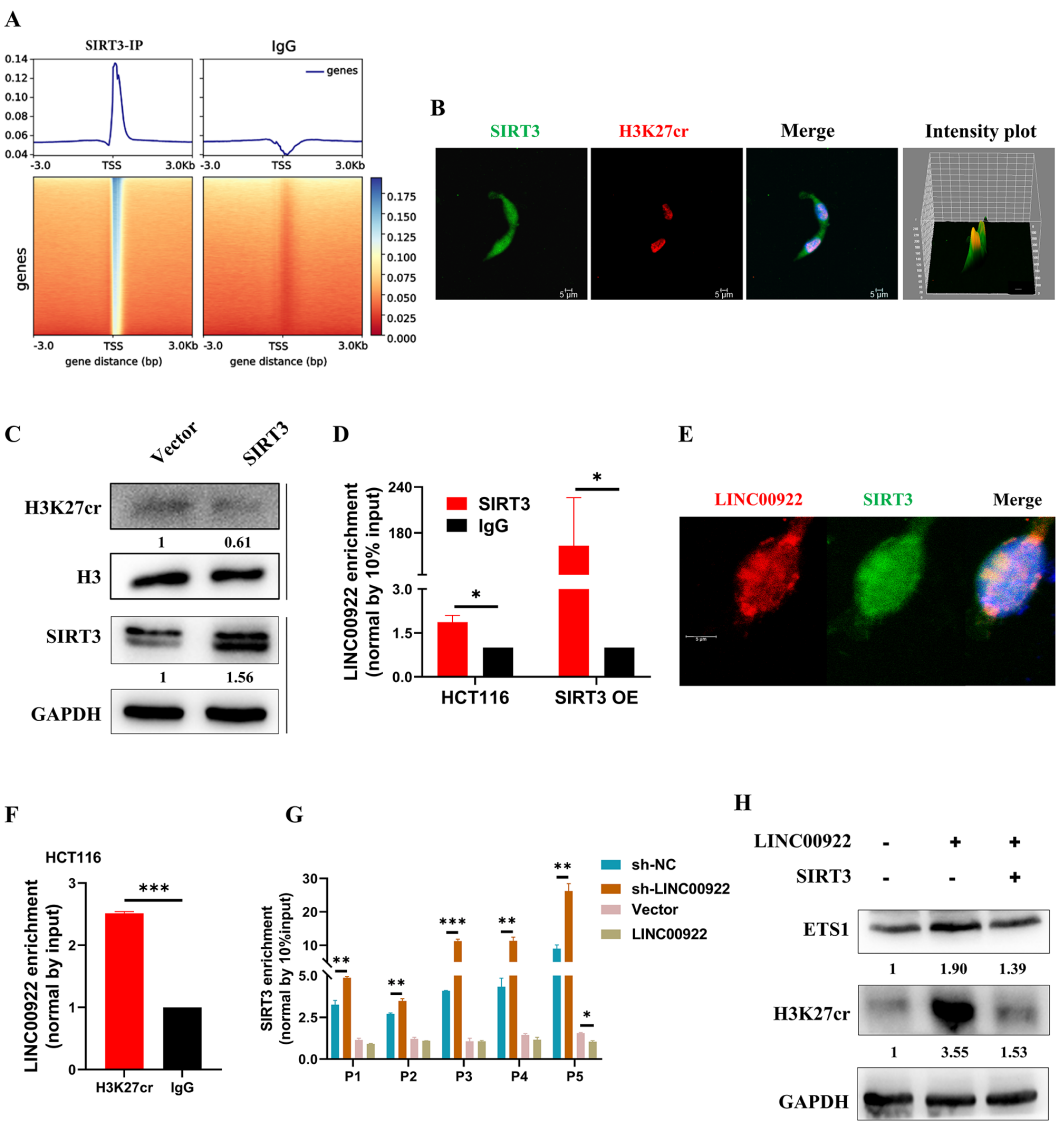
LINC00922 altered H3K27cr occupation via SIRT3. (**A**) Heatmaps and signal density plots centered on transcriptional start sites of genes targeted by SIRT3. (**B**) Immunofluorescence images depicting the location of SIRT3 (green) and H3K27cr (red) in HCT116 cells. Scale bar, 5 μm. (**C**) Immunoblotting analysis of H3K27cr level in HCT116 cells transfected with SIRT3 plasmid for 48 h. (**D**) RIP-qPCR analysis of LINC00922-SIRT3 interaction in HCT116 cells with or without SIRT3 transient overexpression for 48 h (n = 3). (**E**) RNA-FISH and immunofluorescence staining of LINC00922 RNA (red) and SIRT3 protein (green) in HCT116 cells. Scale bar, 5 μm. (**F**) RIP-qPCR analysis of LINC00922-H3K27cr interaction in HCT116 cells. (**G**) ChIP-qPCR analysis of SIRT3 enrichment on the EST1 promoter in HCT116 cells with LINC00922 stable knockdown or transient overexpression for 48 h (n = 3). **H** Immunoblotting analysis of ETS1 expression in HCT116 cells co-transfected with LINC00922 and SIRT3 plasmids for 48 h. The numbers below western bands indicate the ratio of protein versus GAPDH or H3 in experimental groups to that in control group, analyzed using Image J2. Data are represented as means ± SD, *P < 0.05, **P < 0.01, ***P < 0.001, unpaired, two-tailed, Student’s *t*-test

How LINC00922 altered the enrichment level of H3K27cr on the ETS1 promoter was studied. ChIP-seq of HCT116 cells revealed that SIRT3 was distributed near the TSS region (Fig. 7A, Table S6). However, the process by how SIRT3 enriches to the TSS region is yet to be understood. We hypothesized that LINC00922 participated in the enrichment of SIRT3 to the TSS region. To test this, the correlation between SIRT3 and H3K27cr in HCT116 cells was first investigated. Immunofluorescence images showed that SIRT3 and H3K27cr were co-localization in the nucleus of HCT116 cells (Fig. 7B). Expectedly, exogenous SIRT3 reduced the H3K27cr level (Fig. 7C). Next, we studied whether LINC00922 altered H3K27cr occupation by modulating SIRT3. RNA immunoprecipitation (RIP) assay revealed that LINC00922 interacted with SIRT3 in the HCT116 cells, and the enrichment was much greater in HCT116 cells overexpressing SIRT3 (Fig. 7D). Fluorescent in situ hybridization (RNA-FISH) revealed that LINC00922 co-localized with SIRT3 in HCT116 cells (Fig. 7E). The RIP assay also showed that LINC00922 interacted with H3K27cr in HCT116 cells (Fig. 7F). Analysis of the ChIP-seq of SIRT3 found a SIRT3 binding motif, and the promoter region of ETS1 contained sequences matching with this motif (Fig. S5). We subsequently investigated whether LINC00922 influenced the enrichment of SIRT3 on the ETS1 promoter region using ChIP-qPCR. The result revealed that enrichment of SIRT3 in ETS1 promoter region was higher in LINC00922 knockdown cells, whereas exogenous LINC00922 showed the opposite effect (Fig. 7G). Subsequently, a rescue assay was performed to restore the H3K27cr level by co-transfecting with SIRT3 and LINC00922 plasmids into HCT116 cells for 48 h. Notably, overexpression of SIRT3 restored ETS1 expression (Fig. 7H). In summary, our results demonstrated that LINC00922 interacted with SIRT3 and drove it away from the ETS1 promoter region, which increased the H3K27cr level in ETS1 promoter region and activated ETS1 expression (Supplementary Video 1).

## Discussion

Clinical evidence shows variation in Kcr level in different tumors. Previous studies revealed up-regulated Kcr level in cancers, including colon, prostate, thyroid, esophagus, pancreas, and lung neoplasms, and down-regulated Kcr level in cancers, including liver, stomach, and kidney carcinomas [13, 25]. Zhang and colleagues argued that Kcr level was relatively upregulated in liver cancer [8]. In the present investigation, we found higher H3K27cr level in CRC tissues than that in adjacent tissues, and H3K27cr increased with CRC malignancy. Notably, LINC00922 expression in CRC followed a similar trend to H3K27cr. The overexpression of LINC00922 resulted in a significant increase in H3K27cr level. Therefore, it was possible that the overexpression of LINC00922 in CRC tissues led to an increase in H3K27cr modification. Since the high expression of LINC00922 in CRC tissues with distant metastasis or lymph node invasion, it is not surprising that H3K27cr level increases in distant metastasis sites of CRC.

The association between Kcr and cancer metastasis is still poorly understood. Wan et al. discovered a correlation between Kcr level and TNM stage of hepatocellular carcinoma (HCC) and increased Kcr level inhibited HCC cells motility [25]. In contrast, crotonylation of non-histone proteins such as ENO1 at K420 has been shown to accelerate invasion and migration of CRC cells [12]. Moreover, p300-catalyzed crotonylation promotes the invasion and migration of HeLa cells via HNRNPA1 [26], and Kcr promotes migration and invasion of PCa cell lines as well [13]. In the present work, we discovered a positive correlation between the H3K27cr level and the M stage of CRC tissues. The H3K27cr level was significantly higher in distant metastasis tissues than in primary cancer tissues. Intriguingly, NaCr treatment improved CRC cells invasion and metastasis. Genes annotated by ChIP-seq of H3K27cr peaks were found to be involved in metastatic-related pathways. High H3K27cr occupancy on ETS1 promoter transcriptionally activated ETS1 expression, accelerating invasion and metastasis. These findings demonstrated that H3K27cr promoted CRC metastasis.

Most prior investigations have concentrated on the deacetylase activity of SIRT3 rather than its decrotonylase activity. SIRT3 is capable of deacetylating a wide range of substrates, including both histone and non-histone proteins. Multiple studies have proposed the potential recruitment of SIRT3 to specific genomic locations. For instance, Toshinor and colleagues discovered that SIRT3 could regulate gene expression through chromatin localization and interaction with chromatin in defined regions [27]. SIRT3 was recruited to the double strand-break site during DNA repair to promote nonhomologous end-joining repair (NHEJ) by decreasing H3K56ac level [28]. During hepatitis B virus (HBV) infection, nuclear SIRT3 was recruited to the HBV covalently closed circular DNA (cccDNA) to inhibit cccDNA transcription by regulating cccDNA-bound histone acetylation H3 [29]. However, the mechanism underlying SIRT3 recruitment to and release from its target sites remains to be explored. Our findings revealed that the overexpression of SIRT3 significantly reduced the level of H3K27cr, thereby confirming its decrotonylases activity. ChIP-seq analysis demonstrated the enrichment of SIRT3 peaks at TSS sites, which was consistent with H3K27cr peaks. Moreover, LINC00922 was found to interact with SIRT3 in HCT116 cells. However, LINC00922 overexpression blocked SIRT3 recruitment to the ETS1 promoter and then increased ETS1 transcription by increasing H3K27cr level in this region, ultimately promoting cancer metastasis.

## Conclusions

Our study presents a working model for LINC00922-mediated SIRT3 recruitment and H3K27cr modification in human cancer metastasis. Gaining a better understanding of the critical role of histone crotonylation in biological processes could potentially lead to novel cancer treatment strategies.

### Electronic supplementary material

Below is the link to the electronic supplementary material.

Additional file 1: Fig. S1 to Fig. S5

Additional file 2: Table S3

Additional file 3: Table S4

Additional file 4: Table S5

Additional file 5: Table S6

Additional file 6: Supplementary Video 1

## Data Availability

The datasets (TCGA.COAD) analyzed during the current study are available in the UCSC Xena TCGA hub repository (https://xenabrowser.net/hub/). The datasets (GSE 124,540, GSE103479, GSE21510, GSE71222, GSE72970, GSE81582, GSE81986, GSE87211, GSE221575, GSE196964, GSE225857, and GSE96035) analyzed during the current study are available in the GEO database (https://www.ncbi.nlm.nih.gov/gds/).

## Abbreviations

H3K27cr: Crotonylation on Lys27 of histone H3
Kcr: Lysine crotonylation
CRC: Colorectal cancer
GSEA: Gene set enrichment analysis
KEGG: Kyoto encyclopedia of genes and genomes
GO: Gene ontology
ChIP: Chromatin immunoprecipitation
RIP: RNA immunoprecipitation
FISH: Fluorescence in situ hybridization
LncRNA: Long noncoding RNA
DMEM: Dulbecco’s modified eagle medium
GEO: Gene expression omnibus
TCGA: The cancer genome atlas
qRT-PCR: Quantitative real-time PCR
BSA: Bovine serum albumin
EMT: Epithelial-mesenchymal transition
TSS: Transcription start sites
NaCr: Croconic acid disodium salt
SIRT3: Sirtuin 3
