# Supplementary material for: LINC00922 decoys SIRT3 to facilitate the metastasis of colorectal cancer through up-regulation the H3K27 crotonylation of ETS1 promoter
Source: Mol Cancer. 2023 Oct 4;22:163. doi: 10.1186/s12943-023-01859-y (PMC10548613; doi:10.1186/s12943-023-01859-y)
Supplement: Supplementary file 1 — Additional file 1: Fig. S1 to Fig. S5 [file 12943_2023_1859_MOESM1_ESM.docx]

**LINC00922 decoys SIRT3 to facilitate the metastasis of colorectal cancer through up-regulation the H3K27 crotonylation of ETS1 promoter**

Meijian Liao^1^, Xiaolin Sun^1^, Wendan Zheng^1^, Mengdi Wu^1^, Yifan Wang^1^, Jia Yao^1^, Yu Ma^1^, Shoucui Gao^1*^, Dongsheng Pei^1*^

^1^Department of Pathology, Xuzhou Medical University, Xuzhou 221004, P.R. China

Meijian Liao and Xiaolin Sun contribute to this work equally.

**Running title**: H3K27cr promotes colorectal cancer metastasis

**Conflict of interest:** The authors declare no potential conflicts of interest

**Keywords:** Colorectal cancer, metastasis, LINC00922, H3K27cr, SIRT3

***Correspondence:**

dspei@xzhmu.edu.cn (Dongsheng Pei)

gaoshoucui@xzhmu.edu.cn (Shoucui Gao)


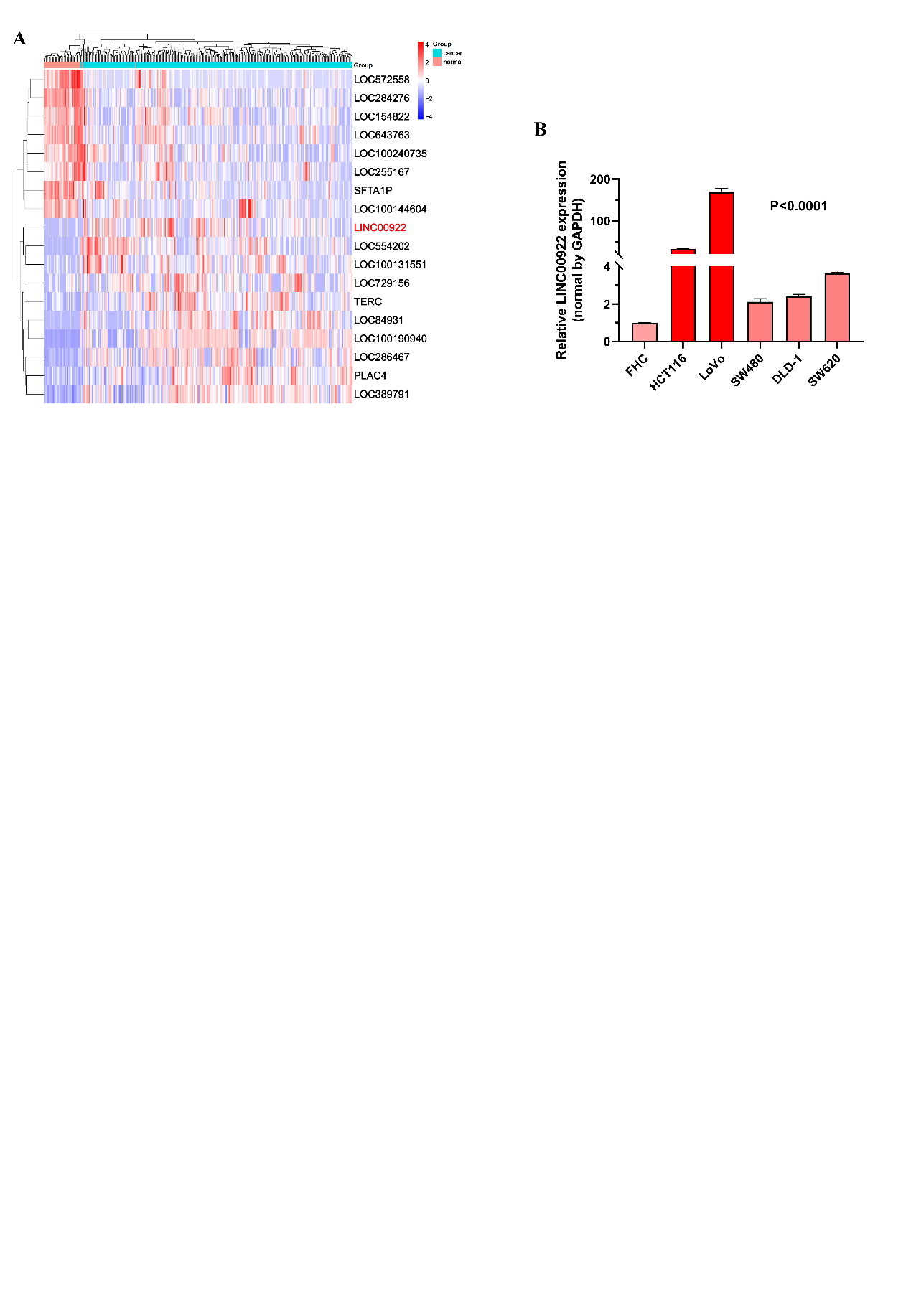


**Fig. S1** High expression of LINC00922. **A.** Heatmap showing expression of lncRNAs between normal and colon cancer tissues of TCGA database. **B.** qRT-PCR analysis of LINC00922 expression across several cell lines. Data are represented as means ± SD, one-way ANOVA test.


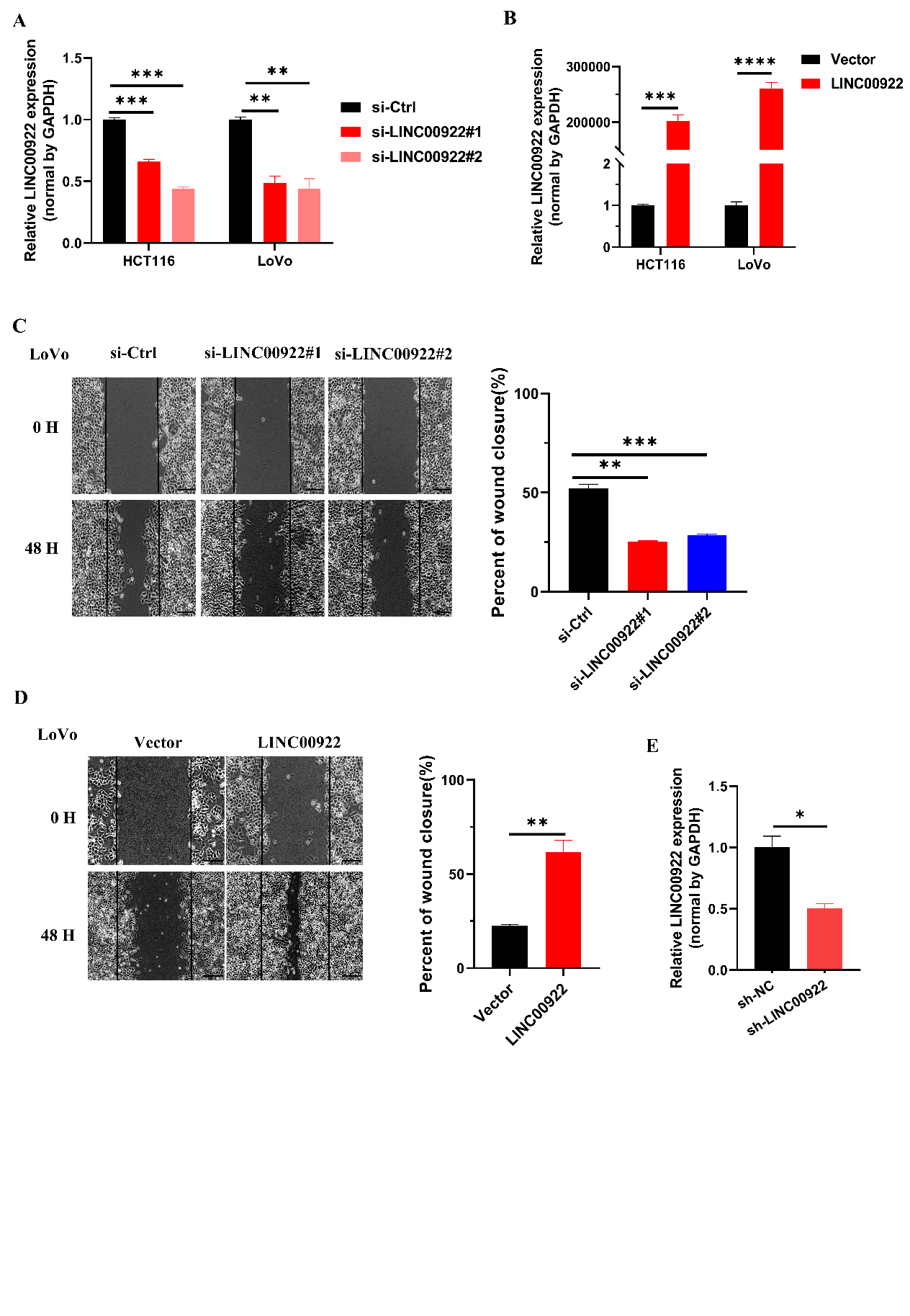


**Fig. S2** LINC00922 promoted LoVo cells motility. **A-B.** qRT-PCR analysis of LINC00922 expression in CRC cells transfected with si-LINC00922 (A) or LINC00922 plasmid (B) for 48 h (n=3). **C-D.** Representative images of wound healing assay showing LoVo cells motility after silence (C) or overexpression (D) of LINC00922 for 48 h (left panel). Scale bar, 200 μm. Cells were counted in 3 random fields (right panel). **E.** qRT-PCR analysis of LINC00922 expression in HCT116 cells with LINC00922 stable knockdown. Data are represented as means ± SD, **P* < 0.05, ***P* < 0.01, ****P* < 0.001, *****P* < 0.0001, unpaired, two-tailed, Student's *t*-test.


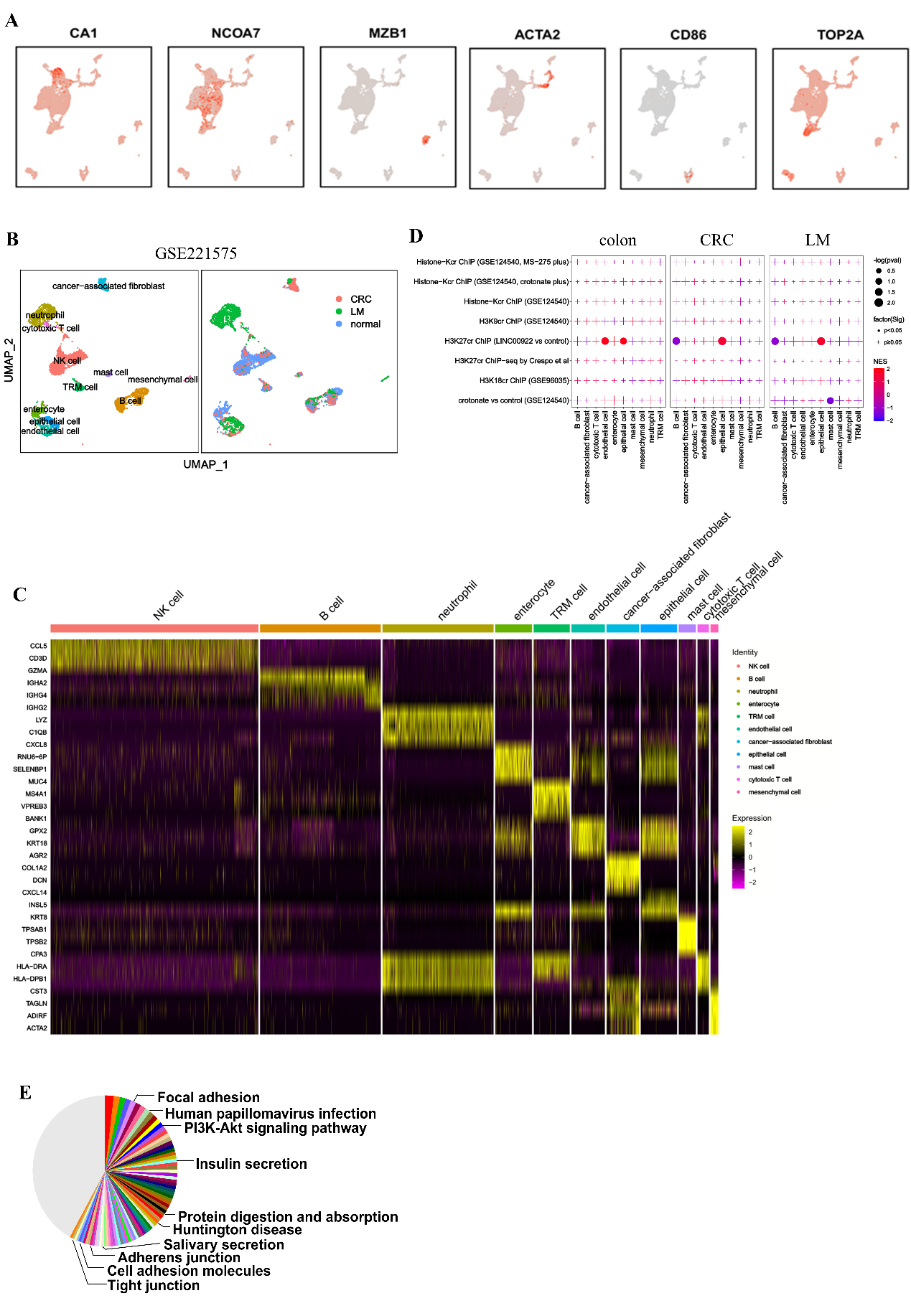


**Fig. S3** Single-cell transcriptomes of CRC tissues. **A.** Scatter plot displaying levels of CA1, NCOA7, MZB1, ACTA2, CD86, and TOP2A in tissues from GSE196964 dataset. **B**. UMAP plotting single cell transcriptome profiles of CRC tissues (GSE221575). The color represents different cell types (left panel) or tissue types (right panel). **C.** Heatmap showing markers of cell types in tissues from the GSE221575 dataset. **D**. Enrichment of 653 genes and other histone crotonylation-related gene sets across various cell types of colon tissues (left panel), CRC tissues (middle panel), and LM (right panel). The significance was calculated using GSEA. Dot indicates P < 0.05, and plus symbol indicates P ≥ 0.05. The red point represents gene set positively expressed in the corresponding cell type, and the blue point represents the opposite. The size of dot represents the negative base 10 logarithm of p-value. **E**. Pie chart showing the biological pathways annotated by 653 genes.


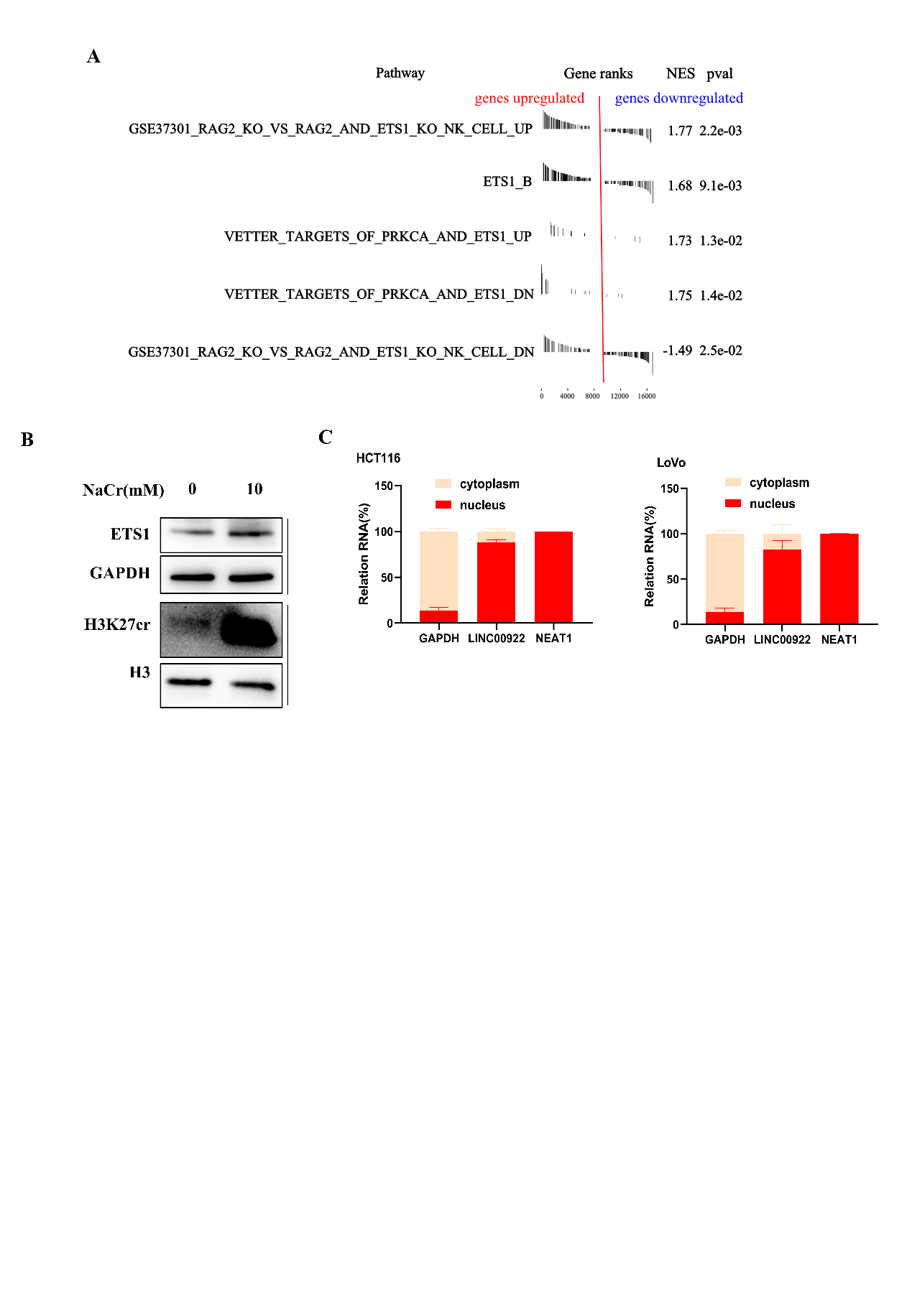


**Fig. S4** The distribution of LINC00922 between cytosol and nucleus, GAPDH and NEAT1 acted as a positive control (n = 3). Chi-square test.


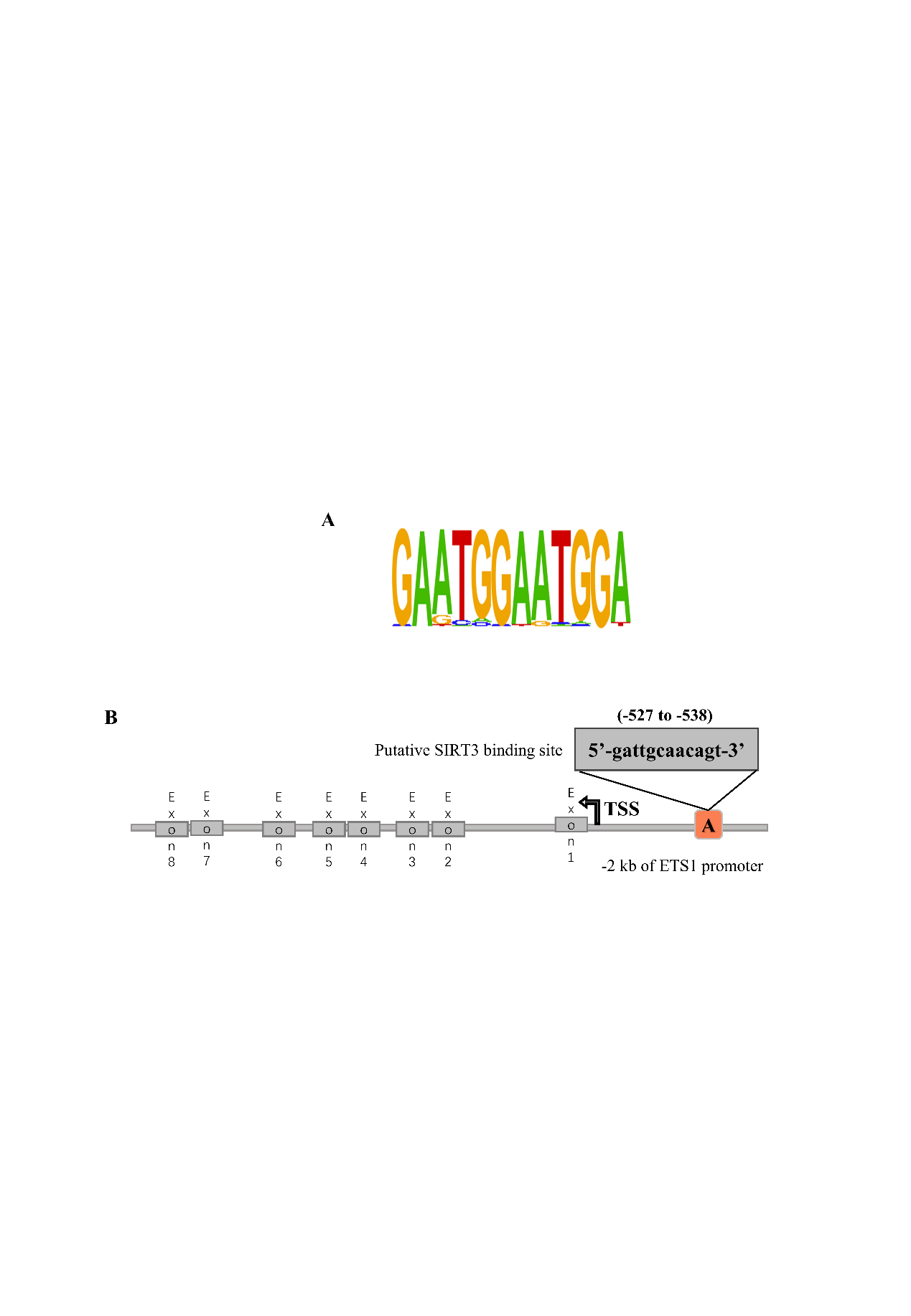


**Fig. S5** Correlation of SIRT3 with *ETS1* promoter. **A.** ChIP-seq analysis of the DNA recognition motif of SIRT3 protein. **B.** A schematic representation of the potential SIRT3 binding sites on the ETS1 promoter. Sequences in the grey box indicate the SIRT3 binding site.

**Table S1** Sequences of siRNAs and shRNA.

| genes | Sequence (5’-3’) |
| --- | --- |
| si-LINC00922#1 | ACGUGGGUGUUGAUUCUAATT |
| si-LINC00922#2 & sh-LINC00922 | GCCUGCACCUACAGAUCUATT |
| si-ETS1 | ACUUGCUACCAUCCCGUACTT |

**Table S2** Primer sequences.

|  | Forward primer (5’-3’) | Reverse primer (5’-3’) |
| --- | --- | --- |
| LINC00922 | GGGAGCAGAATGTACCCCTG | TGCTGTGTTCCCTTTGGGTT |
| ETS1 | TTGAAAGCATAGAGAGCTACGA | CTCTGAGTCGAAGCTGTCATAG |
| GAPDH | GAAGGTGAAGGTCGGAGTC | GAAGATGGTGATGGGATTTC |
| ETS1-P1 | CCTCCTTCAGAAACACACGC | CGTGAGGCATGTGGATGAAG |
| ETS1-P2 | CTTCATCCACATGCCTCACG | AGTCCGTCTGATTCTCCACG |
| ETS1-P3 | TGGAGGCCAGCATTGTTTTC | GGCTCCTGGTCTAAACTCCA |
| ETS1-P4 | GGAGTTTAGACCAGGAGCCA | GCACATTTGAACTCCCAGCA |
| ETS1-P5 | GTCGTGGGAGGGTTGTTAGT | GGACCCCAAACTAGAACCCA |

**The R code of scRNA-seq data processing**

library(Seurat)

library(presto)

library(dplyr)

library(msigdbr)

library(tibble)

library(fgsea)

library(ggplot2)

# Building seurat object of samples from the GSE225857 datasets#

fs=list.files('./GSE_RAW/','^GSM')

fs

library(tidyverse)

samples=str_split(fs,'_',simplify = T)[,1]

lapply(unique(samples),function(x){

y=fs[grepl(x,fs)]

folder=paste0("GS_RAW/", str_split(y[1],'_',simplify = T)[,1])

dir.create(folder,recursive = T)

file.rename(paste0("GSE_RAW/",y[1]),file.path(folder,"barcodes.tsv.gz"))

file.rename(paste0("GSE_RAW/",y[2]),file.path(folder,"features.tsv.gz"))

file.rename(paste0("GSE_RAW/",y[3]),file.path(folder,"matrix.mtx.gz"))

})

samples=list.files("GSE_RAW/")

samples

dir <- file.path('./GSE_RAW',samples)

names(dir) <- samples

counts <- Read10X(data.dir = dir)

scRNA1 = CreateSeuratObject(counts, min.cells=1)

scRNA=subset(scRNA1,subset = nFeature_RNA > 500 & nFeature_RNA < 9000 ) #The parameters for GSE225857 dataset#

scRNA <- NormalizeData(scRNA, normalization.method = "LogNormalize", scale.factor = 10000)

scRNA <- FindVariableFeatures(scRNA, selection.method = "vst", nfeatures = 2000)

all.genes <- rownames(scRNA)

scRNA <- ScaleData(scRNA, features = all.genes)

scRNA <- RunPCA(scRNA, features = VariableFeatures(object = scRNA))

scRNA<- FindNeighbors(scRNA, reduction = "pca", dims = 1:30)

scRNA<- FindClusters(scRNA, resolution = 2)

scRNA<- RunUMAP(scRNA, reduction = "pca", dims = 1:30)

pdf("scRNACluster.pdf",width=9)

DimPlot(scRNA,reduction="umap",label=TRUE)

dev.off()

pdf("scRNASample.pdf",width=9)

DimPlot(scRNA,reduction="umap",group.by="orig.ident")

dev.off()

#Building seurat object of four samples from the GSE221575 dataset. The R code above can't build the seurat object of samples from the GSE221575 dataset.#

seurat1 <- readRDS('GSM6886536.rds')

seurat2 <- readRDS('GSM6886537.rds')

seurat3 <- readRDS('GSM6886538.rds')

seurat4 <- readRDS('GSM6886539.rds')

seurat5 <- readRDS('GSM6886540.rds')

pdf("seurat1-QC.pdf",width=11)

VlnPlot(seurat1, features = c("nFeature_RNA", "nCount_RNA"), ncol = 3)

dev.off()

pdf("seurat2-QC.pdf",width=11)

VlnPlot(seurat2, features = c("nFeature_RNA", "nCount_RNA"), ncol = 3)

dev.off()

pdf("seurat3-QC.pdf",width=11)

VlnPlot(seurat3, features = c("nFeature_RNA", "nCount_RNA"), ncol = 3)

dev.off()

pdf("seurat4-QC.pdf",width=11)

VlnPlot(seurat4, features = c("nFeature_RNA", "nCount_RNA"), ncol = 3)

dev.off()

pdf("seurat5-QC.pdf",width=11)

VlnPlot(seurat5, features = c("nFeature_RNA", "nCount_RNA"), ncol = 3)

dev.off()

mito.genes <- grep(pattern = "^MT-",

x = rownames(seurat1@assays[["RNA"]]),

value = TRUE)

seurat1[["percent.mt"]] <- PercentageFeatureSet(seurat1, pattern = "^MT-")

seurat1 <- subset(seurat1, subset = nFeature_RNA > 200 & nFeature_RNA < 1500 & percent.mt < 15)

mito.genes <- grep(pattern = "^MT-",

x = rownames(seurat2@assays[["RNA"]]),

value = TRUE)

seurat2[["percent.mt"]] <- PercentageFeatureSet(seurat2, pattern = "^MT-")

seurat2 <- subset(seurat2, subset = nFeature_RNA > 200 & nFeature_RNA < 2500 & percent.mt < 15)

mito.genes <- grep(pattern = "^MT-",

x = rownames(seurat3@assays[["RNA"]]),

value = TRUE)

seurat3[["percent.mt"]] <- PercentageFeatureSet(seurat3, pattern = "^MT-")

seurat3 <- subset(seurat3, subset = nFeature_RNA > 200 & nFeature_RNA < 2500 & percent.mt < 15)

mito.genes <- grep(pattern = "^MT-",

x = rownames(seurat4@assays[["RNA"]]),

value = TRUE)

seurat4[["percent.mt"]] <- PercentageFeatureSet(seurat4, pattern = "^MT-")

seurat4 <- subset(seurat4, subset = nFeature_RNA > 200 & nFeature_RNA < 3000 & percent.mt < 15)

mito.genes <- grep(pattern = "^MT-",

x = rownames(seurat5@assays[["RNA"]]),

value = TRUE)

seurat5[["percent.mt"]] <- PercentageFeatureSet(seurat5, pattern = "^MT-")

seurat5 <- subset(seurat5, subset = nFeature_RNA > 200 & nFeature_RNA < 2500 & percent.mt < 15)

seurat <- FindIntegrationAnchors(object.list = list(seurat1, seurat2, seurat3, seurat4, seurat5), dims = 1:30)

seurat <- IntegrateData(anchorset = seurat, dims = 1:30)

seurat <- SCTransform(seurat)

seurat <- RunPCA(seurat, features = VariableFeatures(object =seurat))

pdf("Elbow.pdf")

ElbowPlot(seurat,ndims = 30)

dev.off()

#Building seurat object of all samples from the GSE196964 dataset. There is a big difference in nFeature_RNA values between the four samples of GSE196964. Therefore, the following R code was used.#

seurat1 <- readRDS('GSM5905878.rds')

seurat2 <- readRDS('GSM5905879.rds')

seurat3 <- readRDS('GSM5905880.rds')

seurat4 <- readRDS('GSM5905881.rds')

mito.genes <- grep(pattern = "^MT-",

x = rownames(seurat1@assays[["RNA"]]),

value = TRUE)

seurat1[["percent.mt"]] <- PercentageFeatureSet(seurat1, pattern = "^MT-")

seurat1 <- subset(seurat1, subset = nFeature_RNA>200 & nFeature_RNA < 1000 & percent.mt < 15)

mito.genes <- grep(pattern = "^MT-",

x = rownames(seurat2@assays[["RNA"]]),

value = TRUE)

seurat2[["percent.mt"]] <- PercentageFeatureSet(seurat2, pattern = "^MT-")

seurat2 <- subset(seurat2, subset = nFeature_RNA >200 & nFeature_RNA < 3500 & percent.mt < 15)

mito.genes <- grep(pattern = "^MT-",

x = rownames(seurat3@assays[["RNA"]]),

value = TRUE)

seurat3[["percent.mt"]] <- PercentageFeatureSet(seurat3, pattern = "^MT-")

seurat3 <- subset(seurat3, subset = nFeature_RNA> 200 & nFeature_RNA < 2000 & percent.mt < 15)

mito.genes <- grep(pattern = "^MT-",

x = rownames(seurat4@assays[["RNA"]]),

value = TRUE)

seurat4[["percent.mt"]] <- PercentageFeatureSet(seurat4, pattern = "^MT-")

seurat4 <- subset(seurat4, subset = nFeature_RNA >200 & nFeature_RNA < 6000 & percent.mt < 15)

seurat <- FindIntegrationAnchors(object.list = list(seurat1, seurat2, seurat3, seurat4), dims = 1:30)

seurat <- IntegrateData(anchorset = seurat, dims = 1:50)

seurat <- SCTransform(seurat)

seurat <- RunPCA(seurat, features = VariableFeatures(object =seurat))

pdf("Elbow.pdf")

ElbowPlot(seurat,ndims = 50)

dev.off()

# Integration between samples to remove the batch effect#

scRNA.list <- SplitObject(scRNA, split.by = "orig.ident")

scRNA.list <- lapply(X = scRNA.list, FUN = function(x) {

x <- NormalizeData(x, verbose = FALSE)

x <- FindVariableFeatures(x, verbose = FALSE)

})

features <- SelectIntegrationFeatures(object.list = scRNA.list)

anchors <- FindIntegrationAnchors(object.list = scRNA.list, reduction = "rpca",dims = 1:50,k.filter = 100)

scRNA.integrated <- IntegrateData(anchorset = anchors, dims = 1:30)

scRNA.integrated <- ScaleData(scRNA.integrated, verbose = FALSE)

scRNA.integrated <- RunPCA(scRNA.integrated, verbose = FALSE)

pdf("ElbowIntegrated.pdf")

ElbowPlot(scRNA.integrated,ndims = 30)

dev.off()

scRNA.integrated<- FindNeighbors(scRNA.integrated, reduction = "pca", dims = 1:30)

DefaultAssay(scRNA.integrated)="integrated"

scRNA.integrated<- FindClusters(scRNA.integrated, resolution = 1.5) #The parameters for GSE221517 and GSE196964 datasets#

scRNA.integrated<- FindClusters(scRNA.integrated, resolution = 2) #The parameters for GSE225857 dataset#

scRNA.integrated<- RunUMAP(scRNA.integrated, reduction = "pca", dims = 1:30)

pdf("scRNAIntegratedSample.pdf",width=9)

DimPlot(scRNA.integrated,reduction="umap",group.by="orig.ident")

dev.off()

pdf("scRNAIntegratedCluster.pdf",width=9)

DimPlot(scRNA.integrated,reduction="umap",label=TRUE)

dev.off()

#identification the cell type associated markers#

scRNA.integrated.cellType=subset(scRNA.integrated,idents=c(0:18)) #The parameters for GSE221517 dataset#

scRNA.integrated.cellType=subset(scRNA.integrated,idents=c(0:23)) #The parameters for GSE196964 dataset#

scRNA.integrated.cellType=subset(scRNA.integrated,idents=c(0:33)) #The parameters for GSE225857 dataset#

Idents(scRNA.integrated.cellType)=scRNA.integrated.cellType$integrated_snn_res.1.5 #The parameters for GSE221517 and GSE196964 datasets#

Idents(scRNA.integrated.cellType)=scRNA.integrated.cellType$integrated_snn_res.2 #The parameters for GSE225857 dataset#

scRNA.integrated.celltype.markers <- FindAllMarkers(scRNA.integrated.cellType, only.pos = TRUE, min.pct = 0.25, logfc.threshold = 0.25)

write.table(scRNA.integrated.celltype.markers,file="scRNA.integrated.cellType.markers.txt",sep="\t",quote=F)

scRNA.integrated.celltype.markers %>%

group_by(cluster) %>%

top_n(n = 3, wt = avg_log2FC) -> top3

scRNA.integrated.cellType <- ScaleData(scRNA.integrated.cellType, verbose = FALSE)

pdf("cellTypeMarkerDoHeatmap.pdf",width=20,height=15)

DoHeatmap(scRNA.integrated.cellType, features = top3$gene)

dev.off()

#identification the cell type using CellMarker 2.0 database and following code#

new.cluster.ids <- c("NK cell", "NK cell", "B cell", "B cell", "neutrophil", "neutrophil", "enterocyte", "TRM cell", "endothelial cell", "cancer-associated fibroblast", "NK cell", "epithelial cell", "mast cell", "epithelial cell", "B cell", "NK cell", "neutrophil", "cytotoxic T cell", "mesenchymal cell") #The parameters for GSE221517 dataset#

new.cluster.ids <- c("enterocyte", "epithelial cell", "epithelial cell", "epithelial cell", "colorectal stem cell", "endothelial cell", "colorectal stem cell", "B cell", "colorectal stem cell", "macorphage", "transit-amplifying cell", "enterocyte", "colorectal stem cell", "endothelial cell", "transit-amplifying cell", "cancer-associated fibroblast", "dendritic cell", "cancer-associated fibroblast", "epithelial cell", "NK cell", "NK cell", "enterocyte", "progenitor cell", "mast cell")#The parameters for GSE196964 dataset#

new.cluster.ids <- c("colorectal stem cell", "endothelial cell", "colorectal stem cell", "epithelial cell", "endothelial cell", "neutrophil", "endothelial cell", "enterocyte", "cancer-associated fibroblast", "stromal cell", "stromal cell", "stromal cell", "stem cell", "endothelial cell", "stromal cell", "goblet cell", "neutrophil", "stromal cell", "enterocyte", "epithelial cell", "epithelial cell", "epithelial cell", "stromal cell", "cytotoxic T cell", "goblet cell", "epithelial cell", "macrophage", "enterocyte", "stromal cell", "B cell", "endothelial cell", "goblet cell", "colorectal stem cell", "goblet cell")#The parameters for GSE225857 dataset#

names(new.cluster.ids) <- levels(scRNA.integrated.cellType)

scRNA.integrated.cellType <- RenameIdents(scRNA.integrated.cellType, new.cluster.ids)

scRNA.integrated.cellType$cellType=Idents(scRNA.integrated.cellType)

pdf("scRNA.integrated.cellTypeType.Cluster2.pdf",width=9.5)

DimPlot(scRNA.integrated.cellType,reduction="umap",label=TRUE)&theme(panel.border = element_rect(fill=NA,color="black", size=1.5, linetype="solid"))

dev.off()

pdf("cellTypeMarkerDoHeatmap-celltype.pdf",width=20,height=15)

DoHeatmap(scRNA.integrated.cellType, features = top3$gene)

dev.off()

#fgsea analysis of the association between cell type specific expression and H3K27cr-associated genes#

scRNA.integratedMarker <- wilcoxauc(scRNA.integrated.cellType, 'cellType')

table(scRNA.integratedMarker$group)

for(cluster in unique(scRNA.integrated.cellType$cellType)){

print (cluster)

clusterCell<- scRNA.integratedMarker %>% dplyr::filter(group == cluster) %>% arrange(desc(logFC)) %>% dplyr::select(feature, logFC)

ranks<- deframe(clusterCell)

fgseaRes<- fgseaMultilevel(fgsea_sets, stats = ranks,eps=0, nPermSimple = 10000)

ranks=na.omit(ranks)

fwrite(fgseaRes, file=paste0("/",cluster,".txt",sep=""), sep="\t", sep2=c("", " ", ""))

}

#visualization result of fgsesa using scatter plot#

library(ggplot2)

data.final<-read.csv("GSEA.csv",header=T)

ggplot(data.final,aes(x=celltype,y=pathway,shape=factor(Sig)))+

geom_point(aes(size=`pval`,

color=`NES`))+

theme_bw()+

theme(panel.grid = element_blank(),

axis.text.x=element_text(angle=90,hjust = 1,vjust=0.5))+

scale_color_gradient(low="blue",high="red")+

labs(x=NULL,y=NULL)

#visualization result of fgsesa using heatmap#

library(gplots)

a <- read.csv('heatmap.csv', header = T, check.names = F, row.names = 1)

d <- as.matrix(a)

heatmap.2(d, Colv = F, Rowv = F, col=colorRampPalette(c("brown","white")),colsep=c(1:ncol(d)),rowsep=c(1:nrow(d)), sepcolor = "pink",symkey = F,trace="none")
